# Supplementary material for: Breaking adsorption-energy scaling limitations of electrocatalytic nitrate reduction on intermetallic CuPd nanocubes by machine-learned insights
Source: Nat Commun. 2022 Apr 29;13:2338. doi: 10.1038/s41467-022-29926-w (PMC9054787; doi:10.1038/s41467-022-29926-w)
Supplement: Supplementary file 1 — Supplementary infomation [file 41467_2022_29926_MOESM1_ESM.docx]

**Supplementary Information**

**Breaking Adsorption-Energy Scaling Limitations of Electrocatalytic Nitrate Reduction on Intermetallic CuPd Nanocubes by Machine-Learned Insights**

**Qiang Gao^1,†^, Hemanth Somarajan Pillai^1,†^, Yang Huang^1^,** **Shikai Liu^2^, Qingmin Mu^1^, Xue Han^1^, Zihao Yan^1^, Hua Zhou^3^, Qian He^2,🟉^, Hongliang Xin^1,🟉^ & Huiyuan Zhu^1,🟉^**

^1^Department of Chemical Engineering, Virginia Polytechnic Institute and State University, 635 Prices Fork Rd., Blacksburg, Virginia 24061, USA.

^2^Department of Materials Science and Engineering, National University of Singapore, 9 Engineering Drive 1, Singapore 117575, Singapore.

^3^X-ray Science Division, Advanced Photon Source, Argonne National Laboratory, Lemont, Illinois 60439, USA

**Supplementary Discussion**

**Computational hydrogen electrode under alkaline conditions.** The free energy change of elementary steps in our system requires the calculation of the hydroxyl-electron pair. This is done by using the computational hydrogen electrode (CHE) under alkaline conditions^1^. The alkaline hydrogen evolution reaction and its standard electrode potential are shown below:

$H_{2}O\left( l \right)+e^{-}\leftrightarrow\frac{1}{2}H_{2}\left( g \right)+ OH^{-}\left( \mathrm{aq} \right) U^{0}= -0.83 V$ vs. SHE

The $\Delta G$ for this reaction at a given pH and electrode potential $U_{\mathrm{SHE}}$is given by:

$$\Delta G=.0592\times pH+\mathrm{eU}_{\mathrm{SHE}}=\left( \frac{1}{2}\mu_{H_{2}}+ \mu_{OH^{-}} \right)- (\mu_{H_{2}O}+\mu_{e^{-}})$$

$$\mu_{\mathrm{OH}^{-}}-\mu_{e^{-}}=.0592\times pH+\mathrm{eU}_{\mathrm{SHE}}-\frac{1}{2}\mu_{H_{2}}+\mu_{H_{2}O}$$

The chemical potential of the hydroxyl-electron pair can be expressed at the RHE scale:

$$\mu_{\mathrm{OH}^{-}}-\mu_{e^{-}}=-\frac{1}{2}\mu_{H_{2}}+\mu_{H_{2}O}+\mathrm{eU}_{\mathrm{RHE}}$$

**Calculating Gibbs Free energy of NO_3_^-^_(aq)_ adsorption.** To calculate the free formation energy of *NO_3_, we used a two-step thermodynamic cycle (shown in Supplementary Fig. 8). This avoids the issue of directly calculating the DFT energy for the solvated nitrate anion. The first step is the formation of nitric acid (HNO_3(g)_) from the solvated nitrate anion in alkaline solutions. The second step is the nitrate adsorption with respect to HNO_3(g)_, which can be obtained from DFT calculations with the computational hydrogen electrode model discussed above. The reaction for the first step is written as:

$$N{O_{3}^{-}}_{\left( \mathrm{aq} \right)}+H_{2}O_{(l)}\to HN{O_{3}}_{\left( g \right)}+OH_{\left( \mathrm{aq} \right)}^{-}$$

The $\Delta G^{o}$ value for the above reaction can be calculated based on the corresponding reaction under acidic conditions,

$$N{O_{3}^{-}}_{\left( \mathrm{aq} \right)}+H_{\left( \mathrm{aq} \right)}^{+}\to HN{O_{3}}_{\left( g \right)} \Delta G^{o}= 0.392 eV$$

The $\Delta G^{o}$ value of .392 eV is calculated based on the tabulated values for the Gibbs free energy of formation of HNO_3(l)_ from NO_3_^-^_(aq)_ (0.317 eV) plus the Gibbs free energy of vaporization of HNO_3(l)_ (0.075 eV). Both values can be obtained from the CRC handbook, and the same approach has been done in literature^2-4^. To get the Gibbs free energy of the alkaline reaction at a given pH, an additional correction 0.$059 \times pH$ was added (e.g., 0.392+0.0592×14 = +1.22 eV at pH 14 in our system). The second step considers the nitrate adsorption with respect to HNO_3(g)_:

$$\mathrm{HN}{O_{3}}_{\left( g \right)}+OH_{\left( \mathrm{aq} \right)}^{-}+ * \to^{*}\mathrm{NO}_{3}+H_{2}O_{(l)}+ e^{-}$$

The Gibbs free energy for this reaction ($\Delta G$) can be calculated as:

$$\Delta G=(G_{*NO_{3}}+ G_{H_{2}O_{\left( l \right)}}+\mu_{e^{-}})-(G_{\mathrm{HN}O_{3}\left( g \right)}+\mu_{OH_{(aq)}^{-}}+G_{*})$$

$$=\left( G_{*NO_{3}}+ G_{H_{2}O_{\left( l \right)}} \right)-\left( G_{\mathrm{HN}O_{3}\left( g \right)}+G_{*} \right)+{(\mu}_{e^{-}}-\mu_{OH_{\left( aq \right)}^{-}})$$

The last term was calculated in the previous section while other terms can be calculated from DFT with energy corrections on Supplementary Table 1. Based on the above details the free formation energy for nitrate adsorption can be calculated as:

$\Delta G_{*\mathrm{NO}_{3}}=\Delta G+1.22$.

A similar set of steps can be used to calculate the free formation energies for the other intermediates using NO_3_^-^_(aq)_, OH^-^_(aq)_ and H_2_O_(l)_ as the reference. For free energy profiles of reaction pathways, the GC-DFT was used in order to account for the effects of the applied bias and solvation at the electrode/electrolyte interface. The free formation energies of intermediates on Cu(100) were also calculated using standard DFT and the difference between these two is tabulated in the last column of Supplementary Table 1.

**Scaling relations and activity plot.** Scaling relations were developed to predict free formation energies of reaction intermediates at metal surfaces. Specifically, we use the nitrate and nitrogen adsorption energies relative to NO_3_ and N radicals from regular gas phase DFT calculations without any further corrections as descriptors. In equations 1 and 2, $E_{NO_{3}}^{\mathrm{DFT}}$ and $E_{N}^{\mathrm{DFT}}$ are the DFT energies for NO_3_ and N in a box, $E_{*NO_{3}}^{\mathrm{DFT}}$ and $E_{*N}^{\mathrm{DFT}}$ are the DFT energies for adsorbed nitrate and nitrogen on the surface, and $E_{*}^{\mathrm{DFT}}$ is the DFT energy for the clean surface. Within this definition, a less positive value (or more negative) refers to a stronger adsorption.

$$\Delta E_{*NO_{3}}=E_{*NO_{3}}^{\mathrm{DFT}}-(E_{*}^{\mathrm{DFT}}+E_{NO_{3}}^{\mathrm{DFT}})$$

( 1 )

$$\Delta E_{*N}=E_{*N}^{\mathrm{DFT}}-(E_{*}^{\mathrm{DFT}}+E_{N}^{\mathrm{DFT}})$$

( 2 )

The free formation energies of 7 intermediates were calculated with reference to NO_3_^-^_(aq)_, OH^-^_(aq)_ and H_2_O_(l)_ at 0 V. vs RHE using the same method as in the preceding section. An additional intermediate dependent constant was added to account for the electrolyte/electrode interface and is tabulated in the last column of Supplementary Table 1. We used the *NO_3_ and *N adsorption energies as the descriptors instead of the *NO_3_ and *N formation free energies for consistency with the convention of Bayeschem models. Additionally, this type of descriptors is easier to calculate without bothering with all the free energy corrections in Supplementary Table 1, simplifying the following catalyst screening workflow.

The scaling relations are shown in Supplementary Figure 1 with *NO_3_ and *N adsorption energies as descriptors. Given a point in the descriptor space ($\Delta E_{*NO_{3}}$, $\Delta E_{*N}$), the scaling relations can be used to get the free formation energies and thus the reaction free energies for all the steps within the mechanism. The maximum of the reaction free energies along the reaction pathway at 0 V vs. RHE is then used as the measure of activity at that specific descriptor values in the volcano plot.

**Design of ordered intermetallics with machine learned insights.** Based on our activity heat map (Figure 1), the optimal catalyst for NO3RR should have a stronger adsorption of $NO_{3}^{-}$ and a weaker adsorption of N, i.e., breaking the scaling relation between the free formation energies of *NO_3_ vs. *N. The Bayeschem analysis suggests that one approach to accomplish this is by tuning the active site structure such that the interatomic coupling with metal *d*-states ($\text{V}_{\text{ad}}^{\text{2}}$) increases for *N but stays unaffected for *NO_3_. This can lead to a weaker *N binding energy if the interaction is dominated by repulsive interactions, which is the case for late transition and noble metals. Due to different adsorption configurations of *N and *NO_3_ (hollow and bridge, respectively), this can be realized if an additional neighbor to the hollow *N can be introduced through the subsurface metal layer. With this insight, we have performed detailed structure analysis of 8 (111)- and (100)-like site motifs with different possible bulk phases. We calculated the distance of a hollow *N to the closest subsurface metal atom ($N^{*}-M_{\mathrm{sub}})$ and that to the closest surface atom ($N^{*}-M_{\mathrm{sur}}$). These distances are always calculated based on the lattice constants of the bulk structure and an in-place adsorption of *N. Subsequently the ratio between these two distances was taken and tabulated in the last column of Supplementary Table 3 for different crystal phases and their respective (100)- and (111)-like surfaces. A value of less than one would suggest that the *N is closer to a subsurface metal atom than the surface atom and thus interacts strongly with it, whereas a value of greater than one would mean that the subsurface metal atoms are not directly interacting with the hollow *N. Ideally a surface with a ratio of far less than one is preferred since it could introduce that additional metal neighbor with the largest orbital overlap and thus repulsive interactions, weakening the *N binding energy. We found that only the (100)-surface of the body centered cubic (bcc) phase has this defining feature while all other phases have a theoretical distance ratio $\leq$1 or depending on the c/a ratio for the face centered tetragonal (fct) and the body centered tetragonal (bct) phases.

According to the structural analysis in Supplementary Table 3, we would have to perform a total of 7226 DFT calculations to obtain *N and *NO_3_ binding energies on all (100)- and (111)-like site motifs. However, with the machine learned insights we can reduce that number to ~900 for bcc structures which show the characteristic feature of reduced interlayer distances that is crucial for breaking scaling relations. The materials project API was used to extract bulk structures from the materials project database^5,6^. Specifically, bimetallic (A_3_B and AB) bulk structures where A and B are any of the transition metals were extracted. This resulted in a total of 1545 bulk systems, including 149 fcc and 264 bcc structures (see details in Supplementary Table 3). The atomic simulation environment (ASE)^7^ was then used to create (111)- and (100)-like site motifs from the those bulk structures (see Supplementary Fig. 9). Different surface terminations were considered in generating the screening space. Pymatgen^8^ was then used to find unique hollow and bridge sites for *N and *NO_3_, respectively. We performed DFT calculations to optimize geometries for the clean, *N, and *NO_3_ systems with all 264 bcc structures in order to calculate the adsorption energies and locate them on the activity volcano plot. Calculations were also performed on randomly sampled (100)-terminated face centered cubic (fcc) structures to show that it is not possible to break scaling on this type of structures. In the screening process, the DFT calculations were performed using VASP with the same settings as mentioned previously. Systems were filtered out if surface atoms move more than 1.5 Å to ensure we didn’t have systems with large surface reconstructions.

**
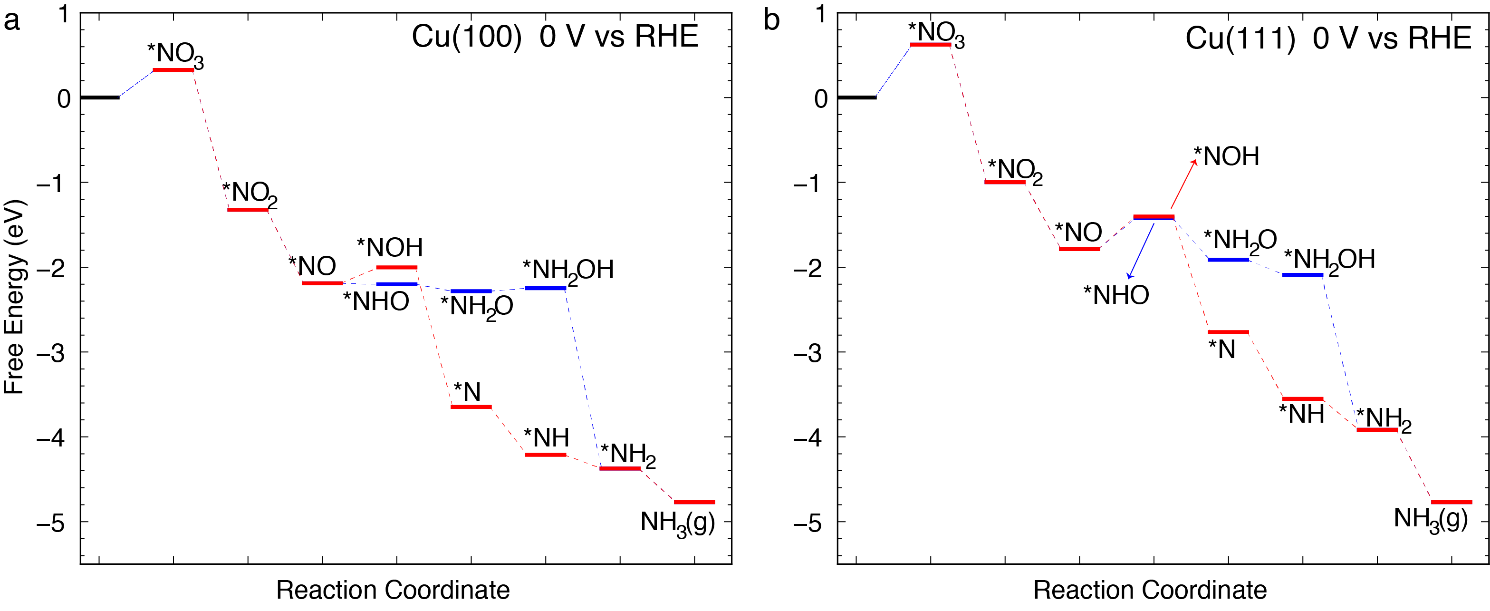
**

**Supplementary Figure 1 | Reaction pathways of NO_3_RR to NH_3_ on Cu(100) and Cu(111).** a, Free energy profile for electrochemical reduction of NO_3_^-^ to NH_3_ on Cu(100) at 0 V vs. RHE from GC-DFT calculations. b, Free energy profile for electrochemical reduction of NO_3_^-^ to NH_3_ on Cu(111) at 0 V vs. RHE from GC-DFT calculations. Two different pathways are shown for both surfaces where the pathways bifurcate after the hydrogenation of *NO.

**Supplementary Figure 2 |** **Linear adsorption-energy scaling relations.** DFT-calculated free formation energies of the 7 reaction intermediates (0 V vs. RHE) in the NO_3_^-^ reduction mechanism as a function of the adsorption energies of the *NO_3_ and *N on fcc(100) (square) and fcc(111) (circle) metal surfaces. Scaling coefficients, intercepts, and R^2^ values are listed within each subplot. The references for the free formation energies are NO_3_^-^ (aq), OH^-^ (aq), and H_2_O(l). For the adsorption energies of *NO_3_ and *N used as reactivity descriptors, NO_3_ and N radicals were used as the reference for convenience and consistency with the convention of Bayeschem models^9^.


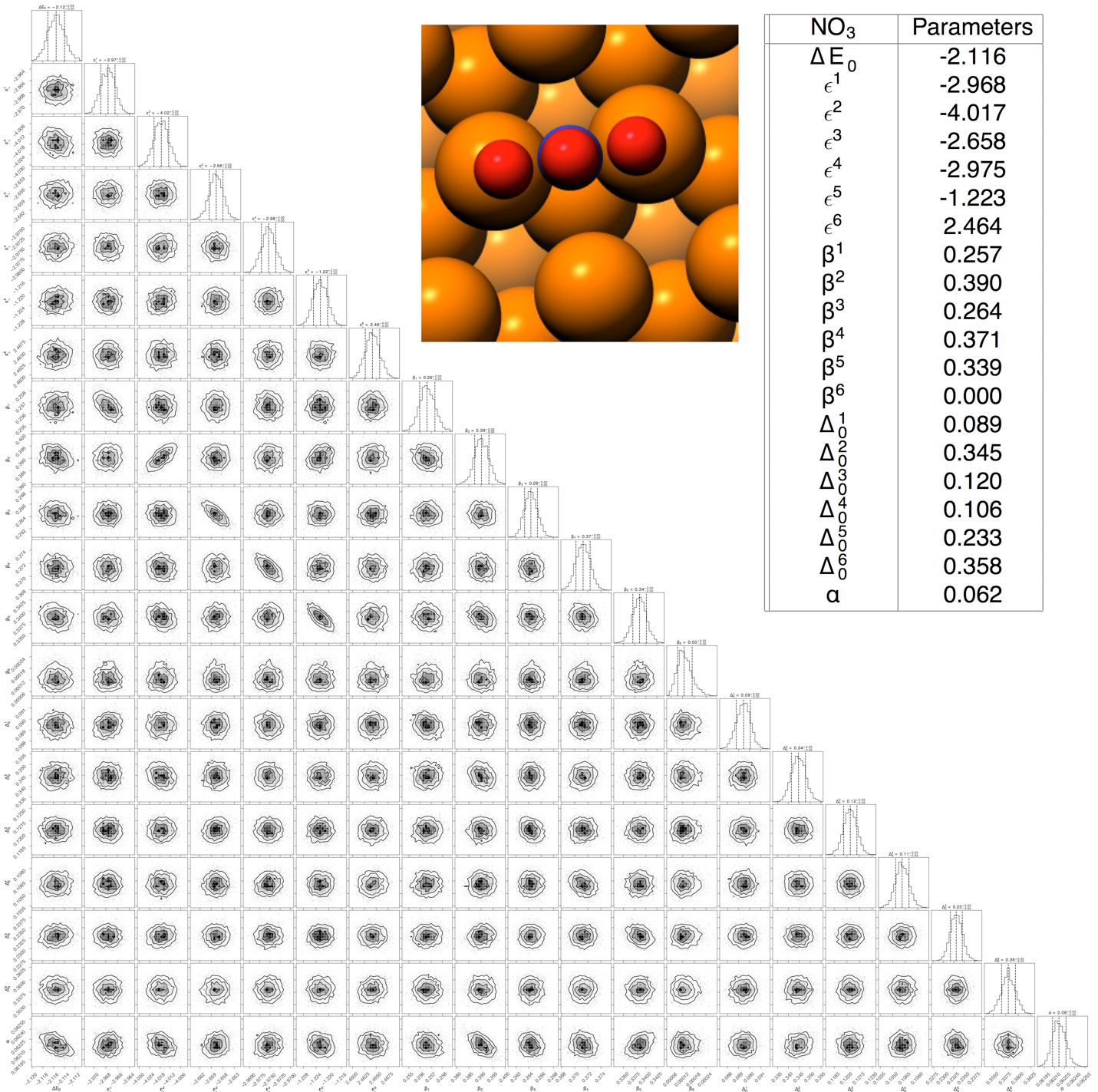
**Supplementary Figure 3 | Bayesian parameterization.** The co-variance of the joint posterior distribution for each pair of parameters and 1D histogram of model parameters ($\Delta E_{0}$, $\epsilon_{a}^{1}$, $\epsilon_{a}^{2}$, $\epsilon_{a}^{3}$, $\epsilon_{a}^{4}$, $\epsilon_{a}^{5}$, $\epsilon_{a}^{6}$,$\beta_{1}$, $\beta_{2}$, $\beta_{3}$, $\beta_{4}$, $\beta_{5}$, $\beta_{6}$, $\Delta_{0}^{1},$ $\Delta_{0}^{2}$, $\Delta_{0}^{3},$ $\Delta_{0}^{4}$, $\Delta_{0}^{5},$ $\Delta_{0}^{6}$, $\alpha$) from MCMC simulations for *NO_3_ adsorption at the bridge site of (111)-terminated fcc metal surfaces. Inset shows a top view of the model structure and a table with the mean value of each parameter.


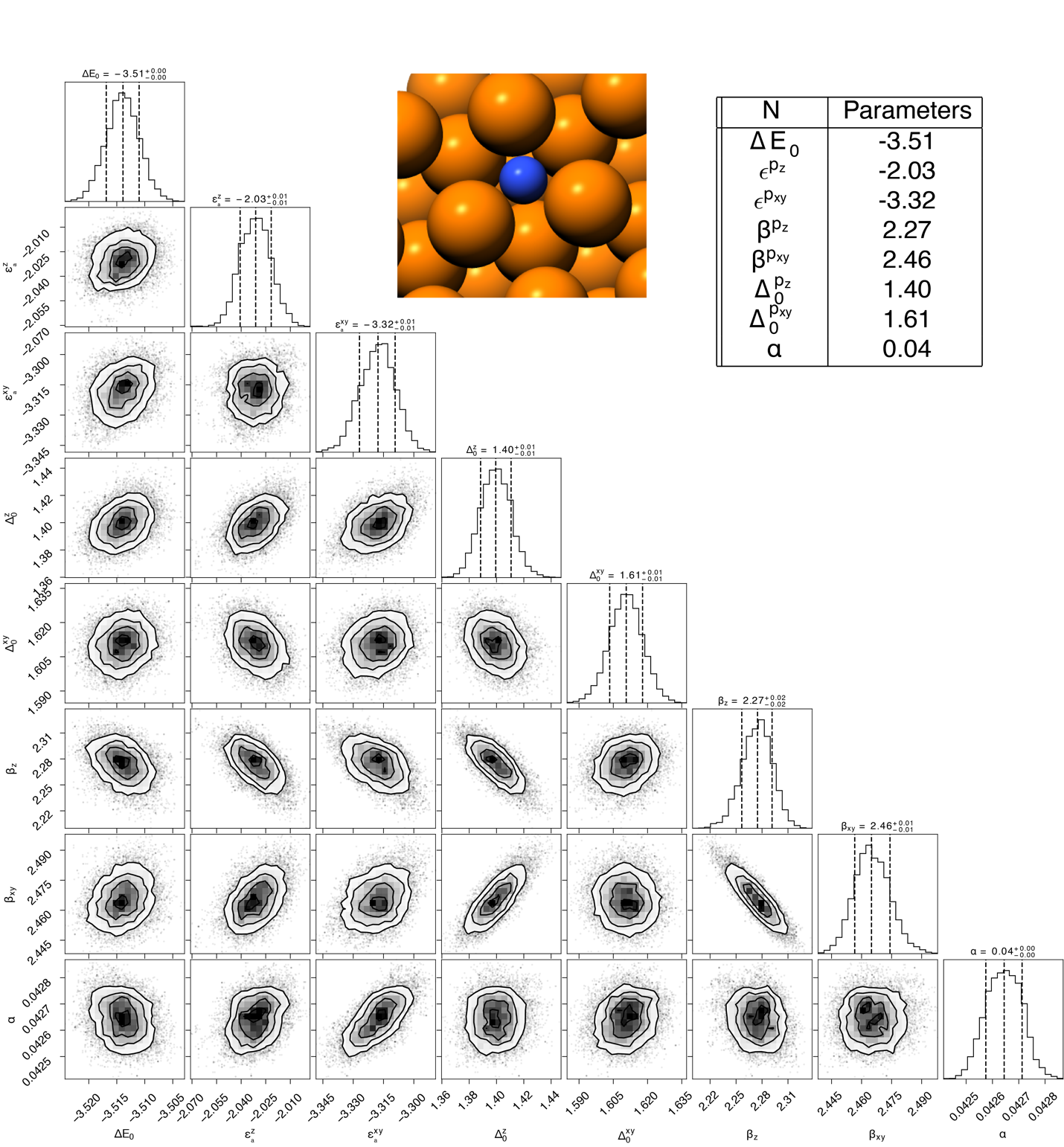


**Supplementary Figure 4 | Bayesian parameterization.** The co-variance of the joint posterior distribution for each pair of parameters and 1D histogram of model parameters ($\Delta E_{0}$, $\epsilon_{a}^{z}$, $\epsilon_{a}^{\mathrm{xy}},$ $\Delta_{0}^{z} ,$ $\Delta_{0}^{\mathrm{xy}}$, $\beta_{z}$, $\beta_{\mathrm{xy}}$, $\alpha$) from MCMC simulations for *N adsorption at the hollow site of (111)-terminated fcc metal surfaces. Inset shows a top view of the model structure and a table with the mean value of each parameter.


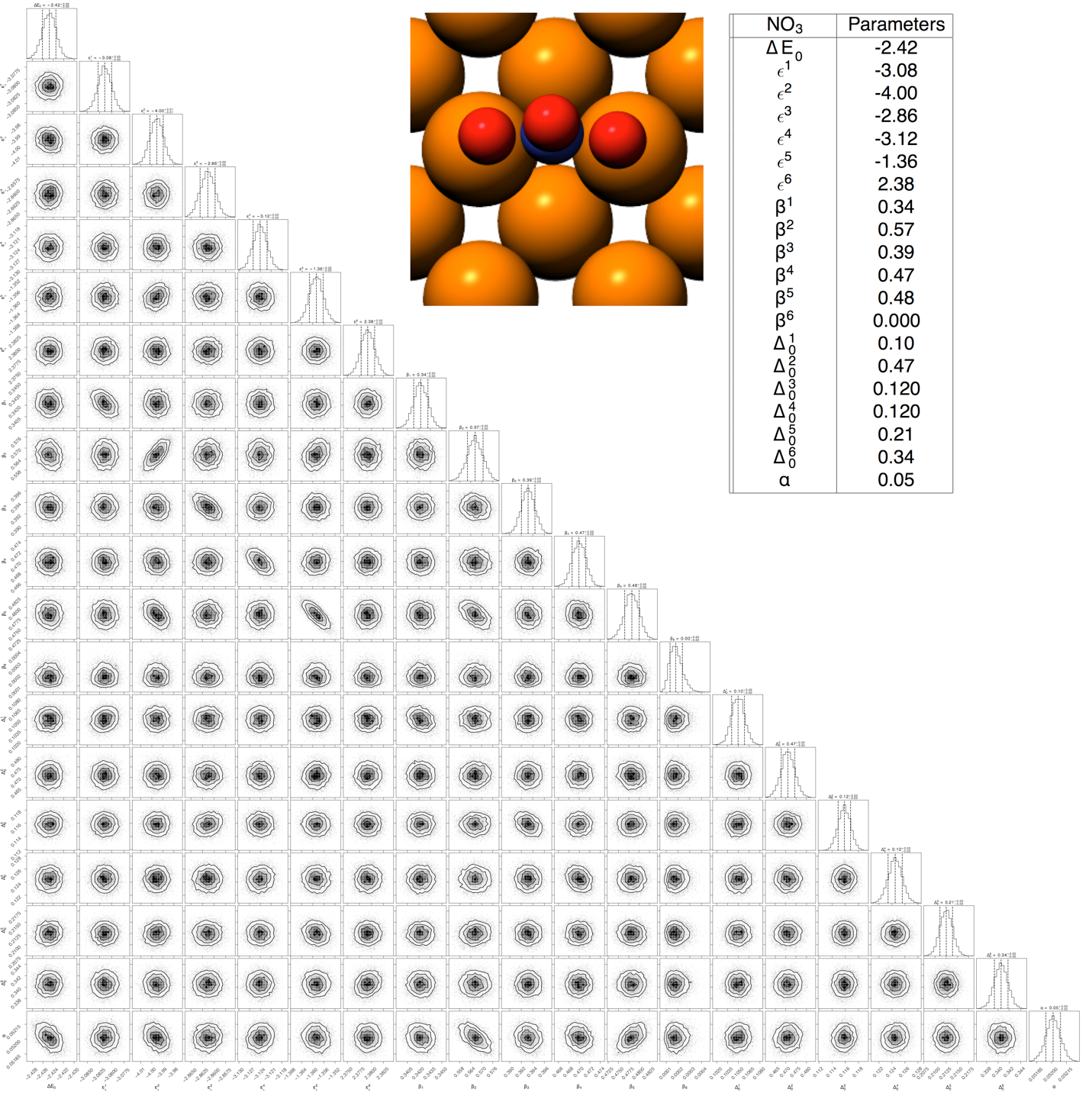
**Supplementary Figure 5 | Bayesian parameterization.** The co-variance of the joint posterior distribution for each pair of parameters and 1D histogram of model parameters ($\Delta E_{0}$, $\epsilon_{a}^{1}$, $\epsilon_{a}^{2}$, $\epsilon_{a}^{3}$, $\epsilon_{a}^{4}$, $\epsilon_{a}^{5}$, $\epsilon_{a}^{6}$,$\Delta_{0}^{1},$ $\Delta_{0}^{2}$, $\Delta_{0}^{3},$ $\Delta_{0}^{4}$, $\Delta_{0}^{5} ,$ $\Delta_{0}^{6}$, $\beta_{1}$, $\beta_{2}$, $\beta_{3}$, $\beta_{4}$, $\beta_{5}$, $\beta_{6}$, $\alpha$) from MCMC simulations for *NO_3_ adsorption at the bridge site of (100)-terminated fcc metal surfaces. Inset shows a top view of the model structure and a table with the mean value of each parameter.


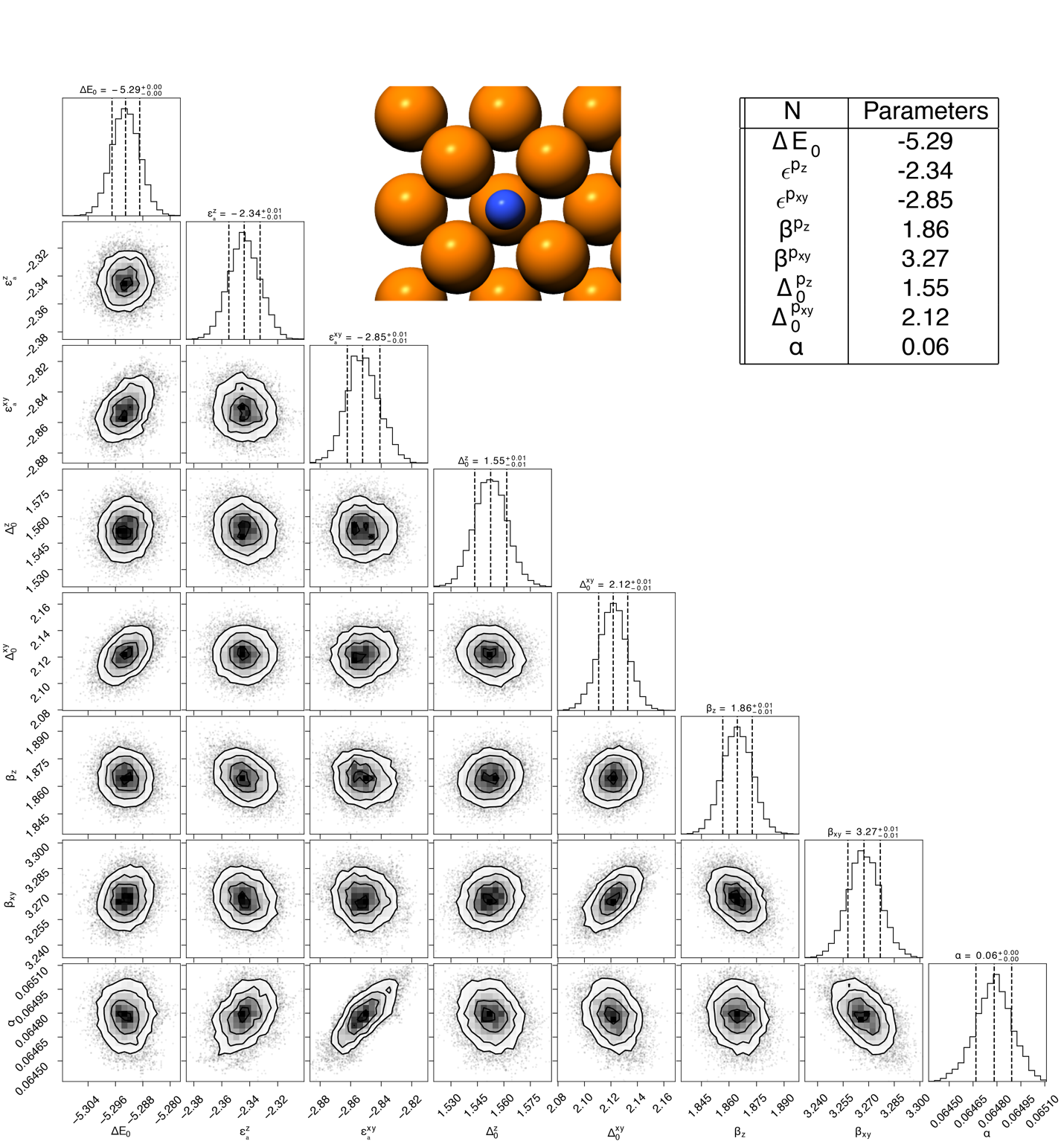


**Supplementary Figure 6 | Bayesian parameterization.** The co-variance of the joint posterior distribution for each pair of parameters and 1D histogram of model parameters ($\Delta E_{0}$, $\epsilon_{a}^{z}$, $\epsilon_{a}^{\mathrm{xy}},$ $\Delta_{0}^{z} ,$ $\Delta_{0}^{\mathrm{xy}}$, $\beta_{z}$, $\beta_{\mathrm{xy}}, \alpha$) from MCMC simulations for *N adsorption at the 4-fold hollow site of (100)-terminated fcc metal surfaces. Inset shows a top view of the model structure and a table with the mean value of each parameter.


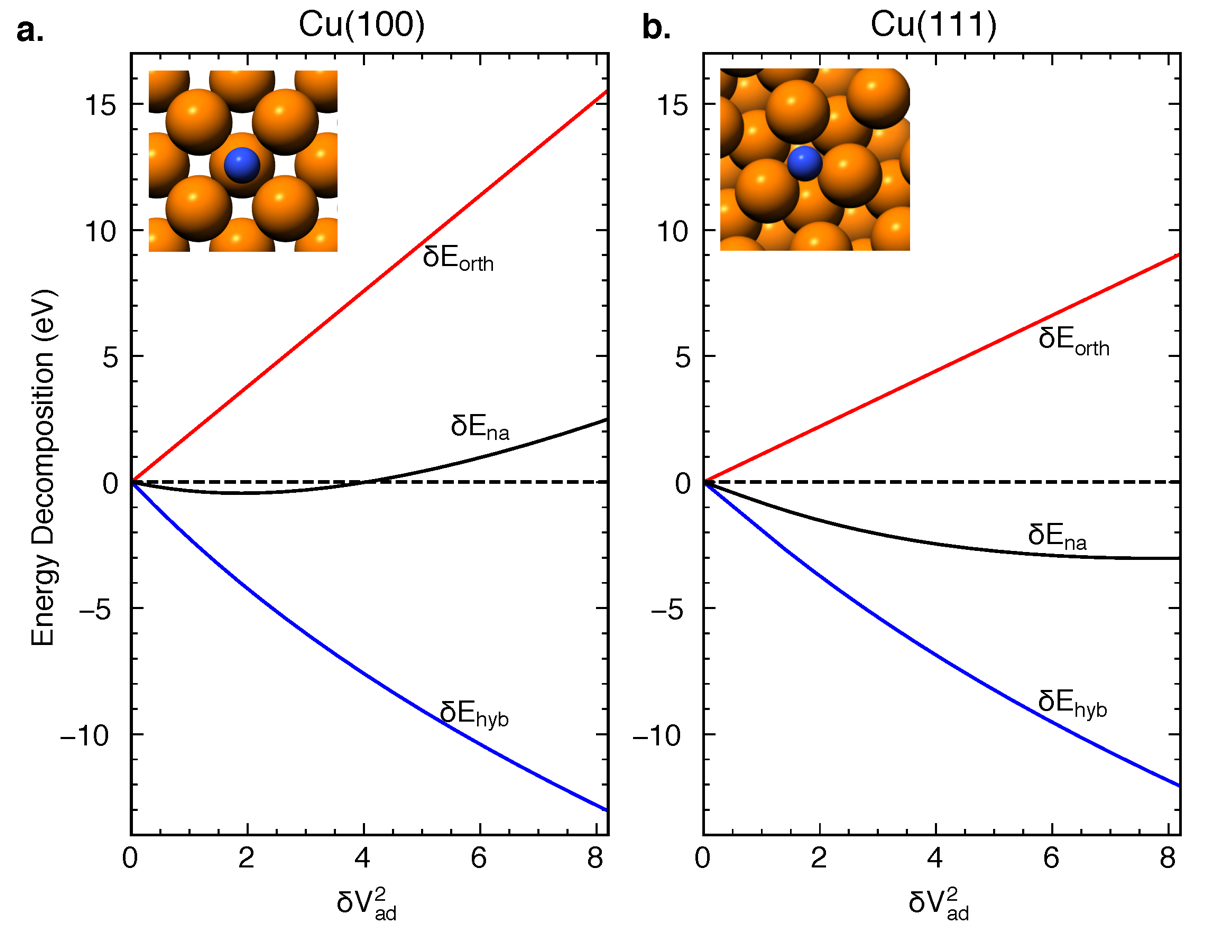


**Supplementary Figure 7 | Physical insights from Bayeschem models.** Decomposition adsorption energies into orbital hybridization and Pauli repulsion from the Bayeschem models of *N at (**a**) Cu(100) and (**b**) Cu(111) as a function of the coupling matrix element squared ($\text{V}_{\text{ad}}^{\text{2}}$). The intercept of the adsorption energy change with 0 line seen in (**a**) represents the switch from hybridization dominated bonding to repulsion on Cu(100) when there is significant orbital overlap $\text{V}_{\text{ad}}^{\text{2}}$, while this switch does not occur on Cu(111) sites in any reasonable $\text{V}_{\text{ad}}^{\text{2}}$.

**
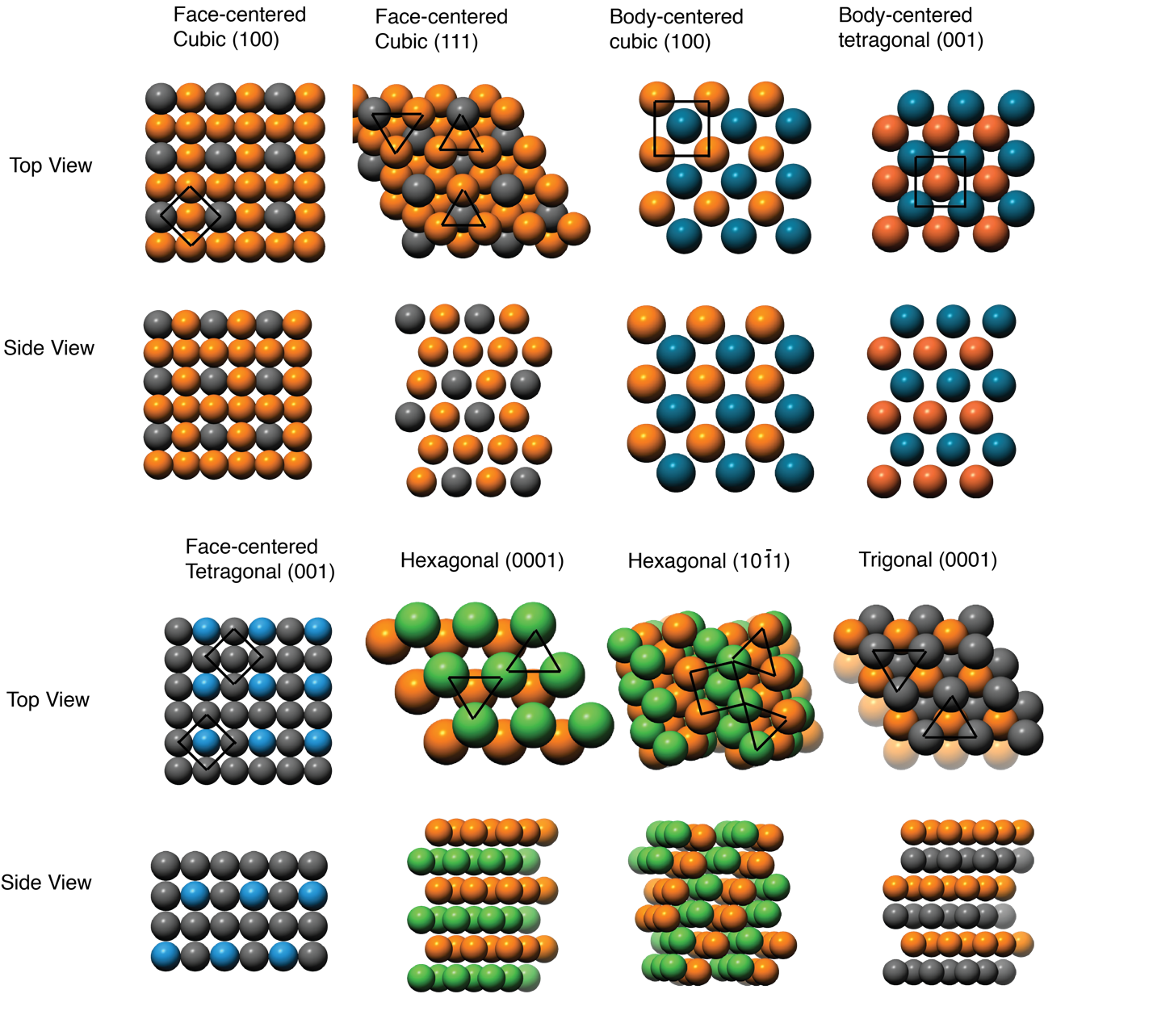
Supplementary Figure 8 | Surface structures of intermetallics.** Top and side views of the (100) and (111)-like site motifs that were considered for structural analysis of stable intermetallic systems from the Materials Project.

**Supplementary Figure 9 | DFT-calculated reaction pathways.** Free energy profiles of NO_3_^-^ reduction to NH_3_(g) on Pd(100), Cu(100), CuPd(100), and RhHf(100) at 0 V vs. RHE (the first element denotes the surface metal). On Pd(100), Cu(100) and CuPd(100), the NO_3_^-^ adsorption is the rate-limiting step whereas on RhHf(100) the *NO hydrogenation to *NHO step is rate limiting.


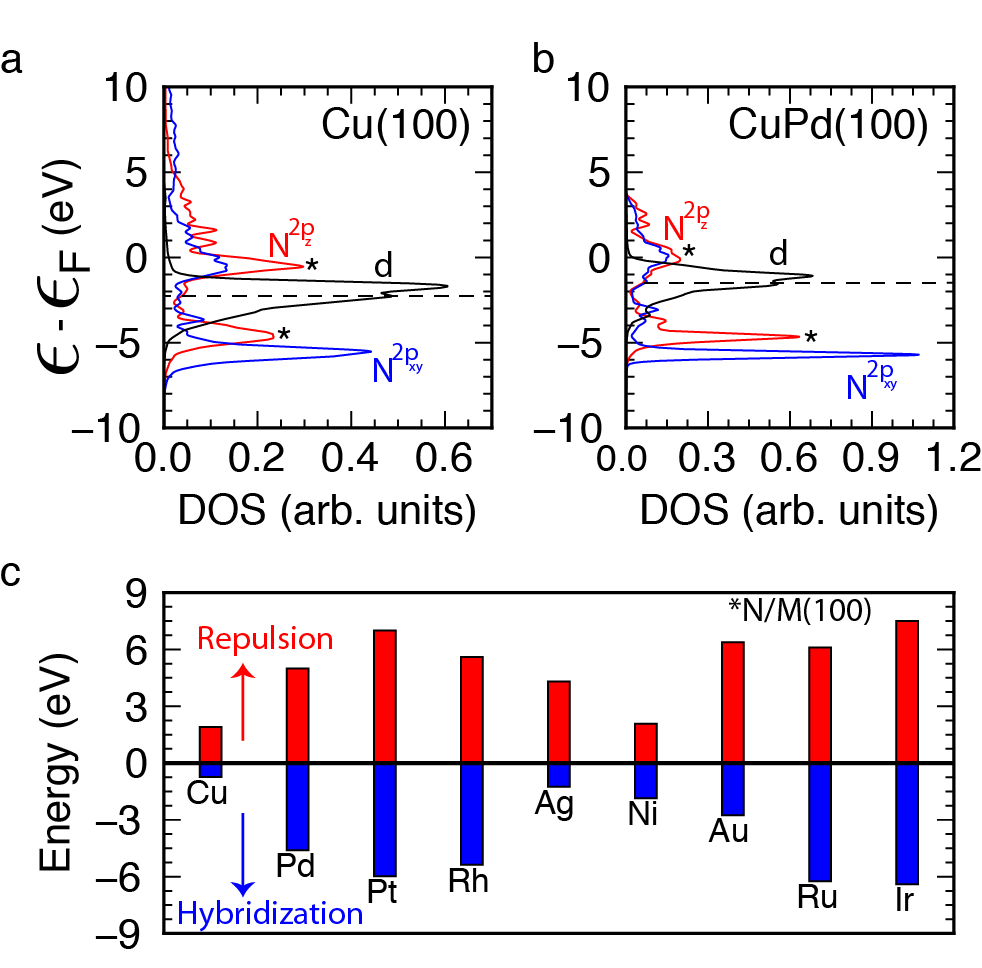


**Supplementary Figure 10 | Electronic structure analysis of *N on Cu(100) and CuPd(100).** **a**, Density of states projected onto the p_xy_ and p_z_ orbitals of *N on Cu(100) and the *d*-states of a surface Cu atom on clean Cu(100). **b**, Density of states projected onto the p_xy_ and p_z_ orbitals of *N on CuPd(100) and the *d*-states of a surface Cu atom on clean CuPd(100). The dashed line represents the *d*-center, and peaks of adsorbate-metal bonding and antibonding states are displayed via markers. **c**, Decomposition of the adsorption energy associated with metal *d*-states into orbital hybridization and repulsion contributions using the *Bayeschem* ML model for *N on M(100) surfaces. The *sp*-band contribution is assumed to be constant in the *Bayschem* ML model.


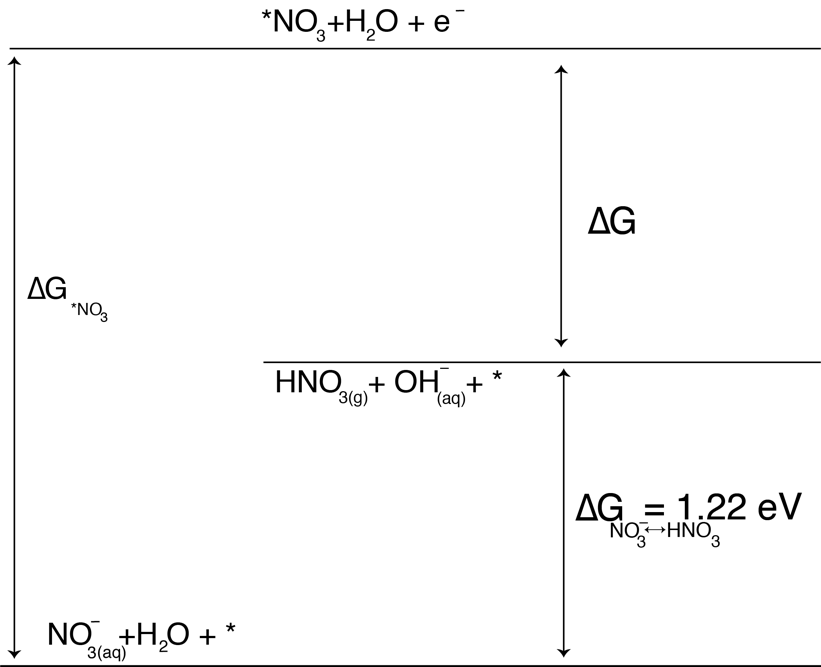


**Supplementary Figure 11 | Thermodynamic cycle used to calculate the Gibbs free energy change of nitrate adsorption.** 1.22 eV was obtained from experimental values from HNO_3_(*l*) formation and free energy of vaporization. $\Delta G$ can be directly calculated from DFT with the computational hydrogen electrode at alkaline conditions.

**Supplementary Table 1 |** **Free energy corrections to adsorbates and gas phase species.** For gas phase species, zero-point energy, enthalpic temperature corrections, and entropy contributions due to vibrational, translational, and rotational degrees of freedom were added. The pressure of HNO_3(g)_, N_2(g)_ and H_2(g)_ was set to 1 bar, and for H_2_O_(g)_ the pressure was set to the water vapor pressure (0.035 bar) at 298 K. For adsorbates, similar corrections were added as well as a constant potential correction which accounts for the effect of solvation and electrochemical bias at the electrode-electrolyte interface. This correction was calculated via grand-canonical DFT simulations for all relevant adsorbates on Cu(100) at 0 V vs. RHE. Further details on these corrections are discussed in the computational details.

| Adsorbate | ZPE (eV) | $\int_{0}^{298} C_{p}\mathrm{dT}$ (eV) | -TS (eV) | Constant potential (eV) |
| --- | --- | --- | --- | --- |
| ^*^NO_3_ | 0.41 | 0.12 | -0.27 | -0.53 |
| ^*^NO_2_ | 0.27 | 0.10 | -0.23 | -0.32 |
| ^*^NO | 0.15 | 0.08 | -0.14 | -0.19 |
| ^*^NHO | 0.48 | 0.06 | -0.10 | -0.13 |
| ^*^NH_2_O | 0.83 | 0.08 | -0.13 | -0.15 |
| ^*^NH_2_OH | 1.12 | 0.11 | -0.23 | -0.14 |
| ^*^NH_2_ | 0.70 | 0.04 | -0.07 | -0.02 |
| HNO_3(g)_ | 0.70 | 0.12 | -0.82 | 0 |
| N_2(g)_ | 0.14 | 0.09 | -0.59 | 0 |
| H_2_O_(g)_ | 0.56 | 0.10 | -0.58 | 0 |
| H_2(g)_ | 0.26 | 0.09 | -0.40 | 0 |

**Supplementary Table 2 | Energy corrections due to the constant electrode potential.** For all adsorbates and selected surfaces, GC-DFT calculations were performed to quantify the effect of solvation and electrochemical bias at 0 V vs. RHE and a pH of 14 on the free formation energies of reaction intermediates. The reported values are the difference in formation free energies calculated using GC-DFT energetics and standard DFT energetics. The last column is the average energy correction from all the surfaces.

| Adsorbate | Pt(100) | Pd(100) | Au(100) | Ag(100) | Cu(100) | Average (eV) |
| --- | --- | --- | --- | --- | --- | --- |
| *NO_3_ | 0.416 | 0.632 | 0.539 | 0.726 | 0.529 | 0.568 |
| *NO_2_ | 0.262 | 0.370 | 0.487 | 0.378 | 0.320 | 0.363 |
| *NO | 0.005 | 0.012 | 0.054 | 0.078 | 0.192 | 0.068 |
| *NHO | -0.196 | -0.052 | 0.289 | 0.129 | 0.128 | 0.060 |
| *NH_2_O | -0.174 | 0.030 | 0.127 | 0.164 | 0.148 | 0.059 |
| *NH_2_OH | -.224 | -.007 | 0.020 | 0.193 | 0.137 | 0.024 |
| *NH_2_ | -.306 | -.120 | -.102 | 0.022 | 0.02 | -.097 |

**Supplementary Table 3 | Free formation energies at 0 V vs. RHE for Cu(100) and Cu(111).** Free formation energies (in eV) were calculated with respect to NO_3_^-^ (aq), OH^-^ (aq), and H_2_O(l) using GC-DFT energies and corrections from Supplementary Table 1.

| Adsorbate | Cu(100) | Cu(111) |
| --- | --- | --- |
| *NO_3_ | 0.325 | 0.624 |
| *NO_2_ | -1.322 | -0.997 |
| *NO | -2.190 | -1.786 |
| *NHO | -2.201 | -1.422 |
| *NH_2_O | -2.283 | -1.912 |
| *NH_2_OH | -2.248 | -2.091 |
| *NH_2_ | -4.374 | -3.917 |

**Supplementary Table 4 | Structural analysis of the design space of ordered intermetallics.** Different (100) and (111)-like site motifs were considered for structural analysis of stable intermetallic systems from the Materials Project. The number of systems that would be required for DFT calculations is listed in columns 3 to 5 for each facet and phase. This considers the different surface terminations and adsorption sites. The last column tabulates the theoretical ratio between the distance of a *N to the subsurface metal and the distance between a *N to a surface metal atom. The bottom row is the total number of clean, *N, and *NO_3_ calculations that would be required if we sample the whole design space directly using DFT calculations.

| **Crystal Phase** | **Surface** | **Number of clean systems** | **Number of *N systems** | **Number of *NO_3_ systems** | $\frac{\mathbf{N}^{\mathbf{*}}\mathbf{-}\mathbf{M}_{\mathbf{sub}}\boldsymbol{(Å)}}{\mathbf{N}^{\mathbf{*}}\mathbf{-}\mathbf{M}_{\mathbf{sur}}\boldsymbol{(Å)}}$ |
| --- | --- | --- | --- | --- | --- |
| Face centered cubic | (100) | 298 | 447 | 298 | $1$ |
|  | (111) | 149 | 596 | 447 | $\surd2$ |
| Body centered cubic | (100) | 264 | 319 | 264 | $\frac{1}{\surd2}$ |
| Hexagonal | (10-11) | 116 | 116 | 580 | $1$ |
|  | (0001) | 116 | 232 | 116 | $\frac{\sqrt{3}c}{2a}$ |
| Face centered tetragonal | (001) | 176 | 264 | 176 | $\frac{c}{a}$ |
|  | (010) | 88 | 264 | 264 | $\frac{a}{min(c, a)}$ |
|  | (112) | 88 | 352 | 264 | $\surd2$ |
| Body centered tetragonal | (001) | 68 | 68 | 68 | $\frac{c}{\sqrt{2}a}$ |
| Trigonal | (0001) | 182 | 364 | 182 | $\frac{\sqrt{3}c}{2a}$ |
| Total Number of systems | | 1545 | 3022 | 2659 |  |


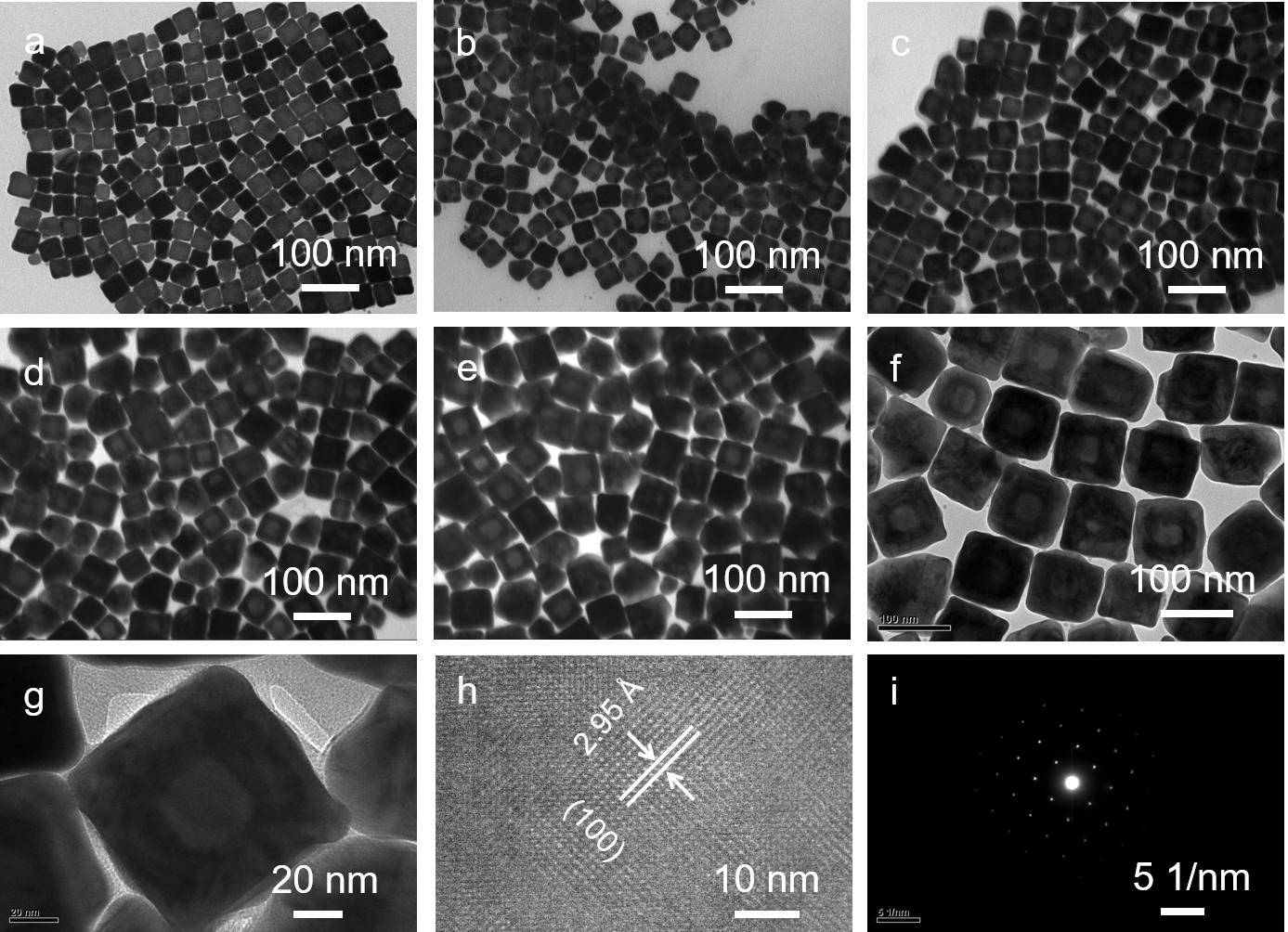


**Supplementary Figure 12 | Characterizations of the ordered CuPd nanocubes.** TEM images of synthesized ordered CuPd nanocubes obtained with different amount 1,2-TDD. **a**, 0.1 mmol, **b**, 0.2 mmol, **c**, 0.4 mmol, **d**, 0.6 mmol, **e**, 0.8 mmol, **f**, 1 mmol. **g**, A TEM image of a single ordered hollow CuPd nanocubes in (f). **h**, An HRTEM image of a typical ordered hollow CuPd nanocube. **i**, A representative SAED pattern of a single ordered hollow CuPd nanocube.

We designed and conducted a series of control experiments to explore optimized experimental parameters to form uniform and high-quality ordered intermetallic CuPd nanocubes (Supplementary Fig. 12). By adjusting the amount of TDD, we can effectively tune the size of the ordered CuPd nanocubes. For instance, by adding 0.1 mmol TDD into the reaction system, only solid ordered CuPd nanocubes were obtained (Supplementary Fig. 12a). With the increase of the amount of TDD, the size of the ordered CuPd nanocubes increases, and the hollow structure becomes more evident. When the amount of TDD was increased to 1 mmol, the size of the ordered hollow CuPd nanocubes became about 100 nm (Supplementary Fig. 12f, 12g). The HRTEM image (Supplementary Fig. 12h) also shows clear lattice fringes with a lattice spacing of 2.95 Å, and the SAED pattern of the single ordered hollow CuPd nanocube depicted in Supplementary Fig. 12i indicated that the ordered hollow CuPd nanocube is single crystalline.


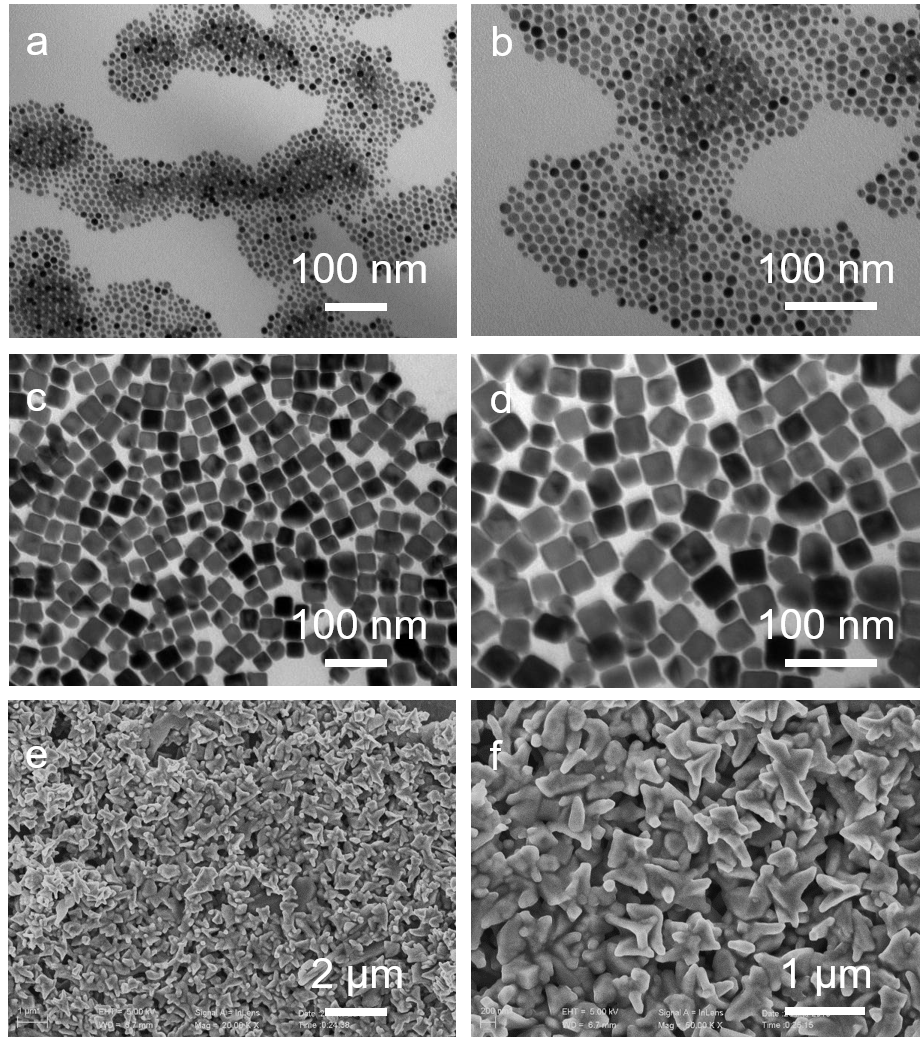


**Supplementary Figure 13 | TEM images. a**, **b**, TEM images of the products synthesized with the addition of 0.1 mL TOP. **c**, **d**, TEM images of the products synthesized with the addition of 0.25 mL TOP. **e**, **f**, SEM images of the products synthesized with the addition of 1 mL TOP.

The amount of TOP also plays an important role in the successful ordered CuPd nanocube preparation (Supplementary Fig. 13). Decreasing the amount of TOP to 0.1 mL leads to the formation of smaller spherical nanoparticles, while increasing the amount of TOP to 1 mL, we obtained larger dendrites.


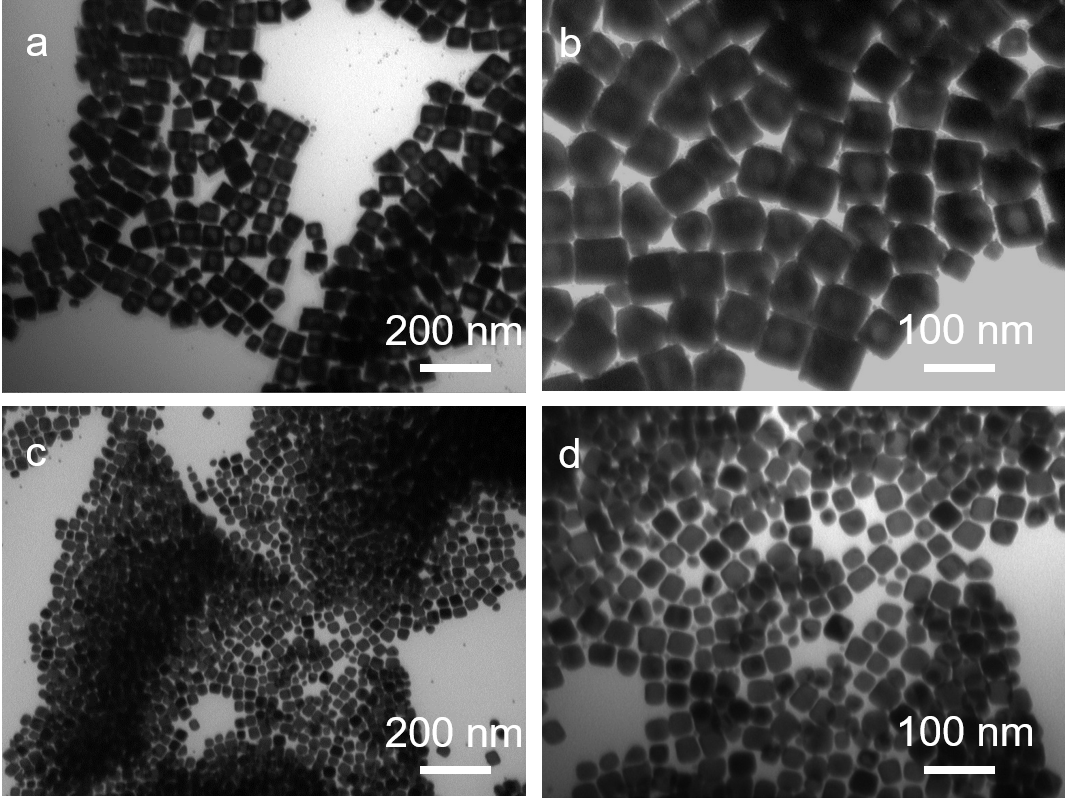


**Supplementary Figure 14 | TEM images of products obtained at different temperature**. **a**, **b**, 230 ^o^C; **c**, **d**, 280 ^o^C.

We also studied the effects of temperature (Supplementary Fig. 14). When the temperature decreased to 230 ^o^C, larger hollow nanocubes were obtained, while increasing the temperature to 280 ^o^C, smaller solid nanocubes were synthesized.


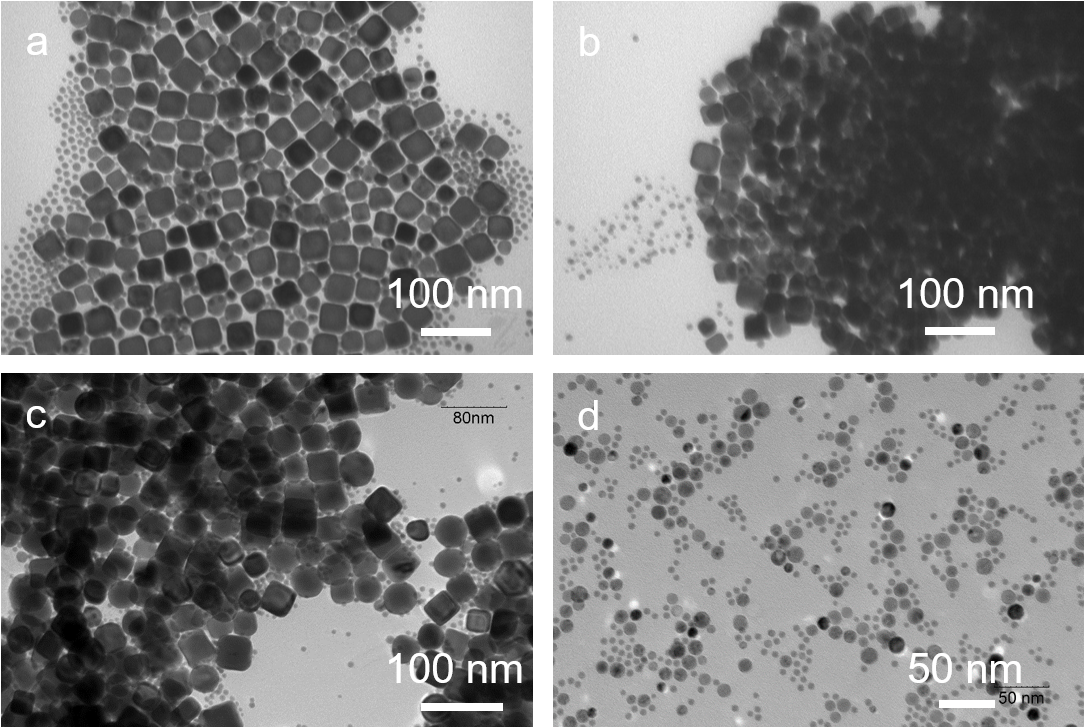


**Supplementary Figure 15 | TEM images of the products synthesized with different Pd:Cu radio**. **a**, 3:1; **b**, 2:1; **c**, 1:2 and **d**, 1:3.

The ratio of Pd/Cu plays a key role in the synthesis process (Supplementary Fig. 15). When the molar ratio of Pd/Cu was changed to 3/1, 2/1 or 1/2, a mixture of nanocubes with smaller spherical nanoparticles was obtained. When the ratio of Pd/Cu was changed to 1/3, only spherical nanoparticles were obtained.


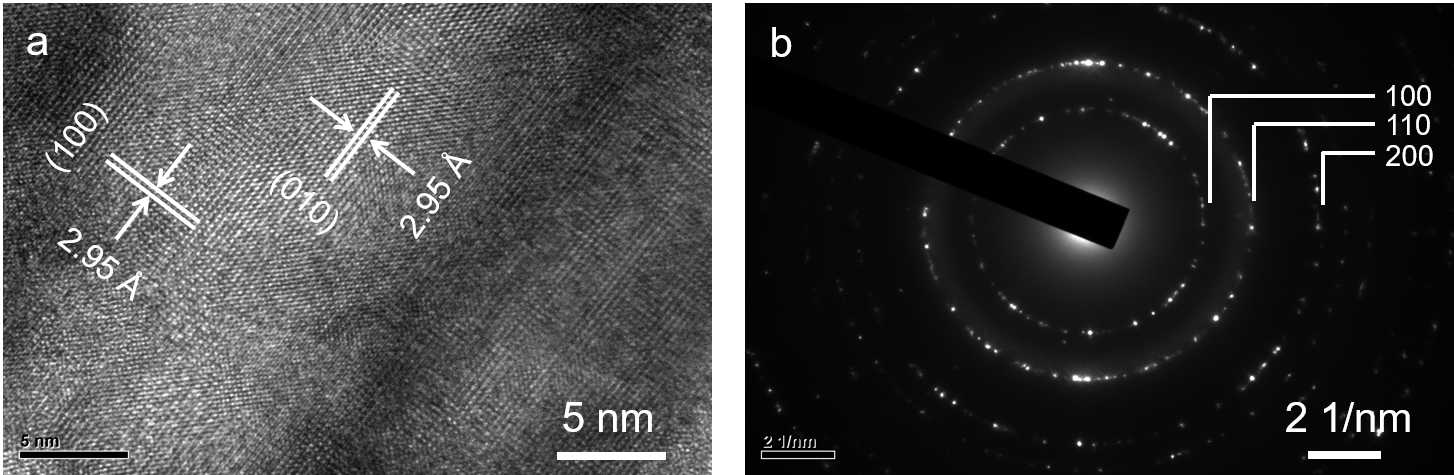


**Supplementary Figure 16 | HRTEM characterization.** **a,** An HRTEM image of ordered CuPd nanocubes. **b,** The selected area electron diffraction (SAED) patterns of the ordered CuPd nanocubes.


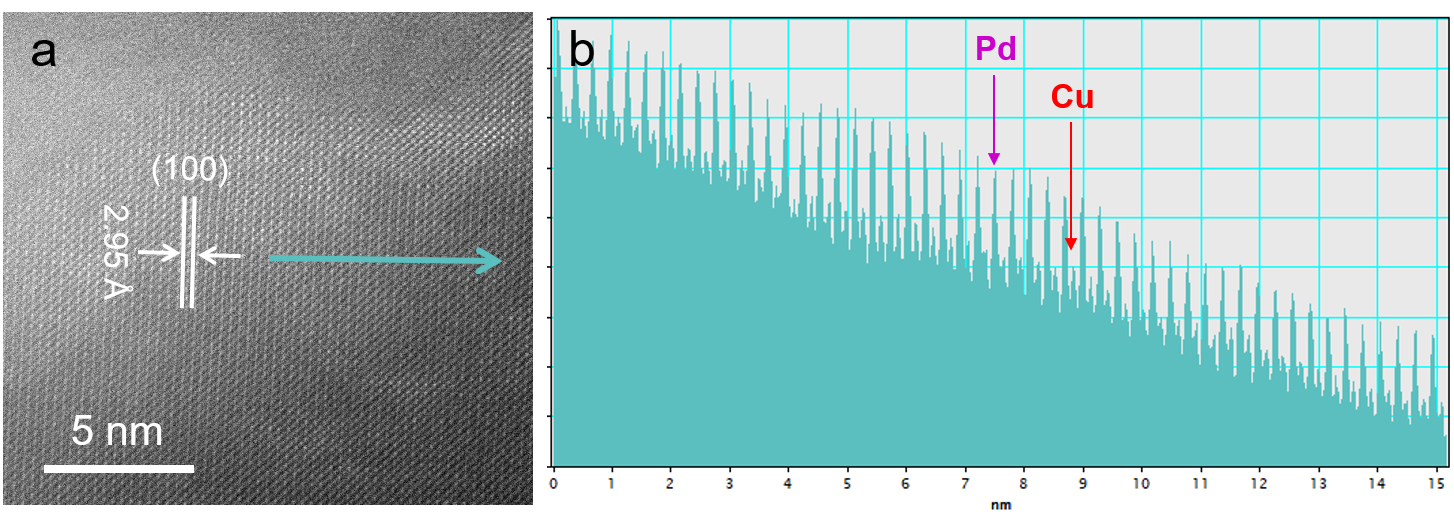


**Supplementary Figure 17 | STEM characterization.** **a,** An atomic-resolution HAABF-STEM image of the ordered CuPd nanocube. The cyan arrow indicates the line scan direction. **b**, Corresponding line scan profile across the line scan position shown in **a**.

**Supplementary Figure 18 | EDX characterization.** EDX spectrum of synthesized ordered CuPd nanocubes.


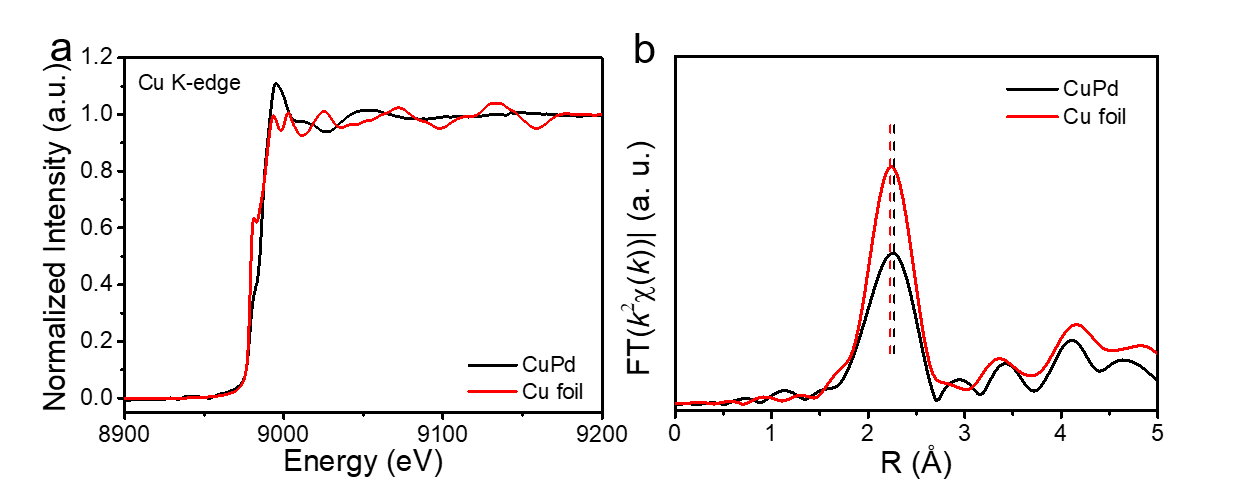


**Supplementary Figure 19 | XAFS spectra.** **a,** Cu K-edge XANES spectra of ordered CuPd nanocubes and Cu foil reference. **b,** EXAFS Fourier transformed k^2^-weighted χ(k) function spectra of ordered CuPd nanocubes and Cu foil reference.


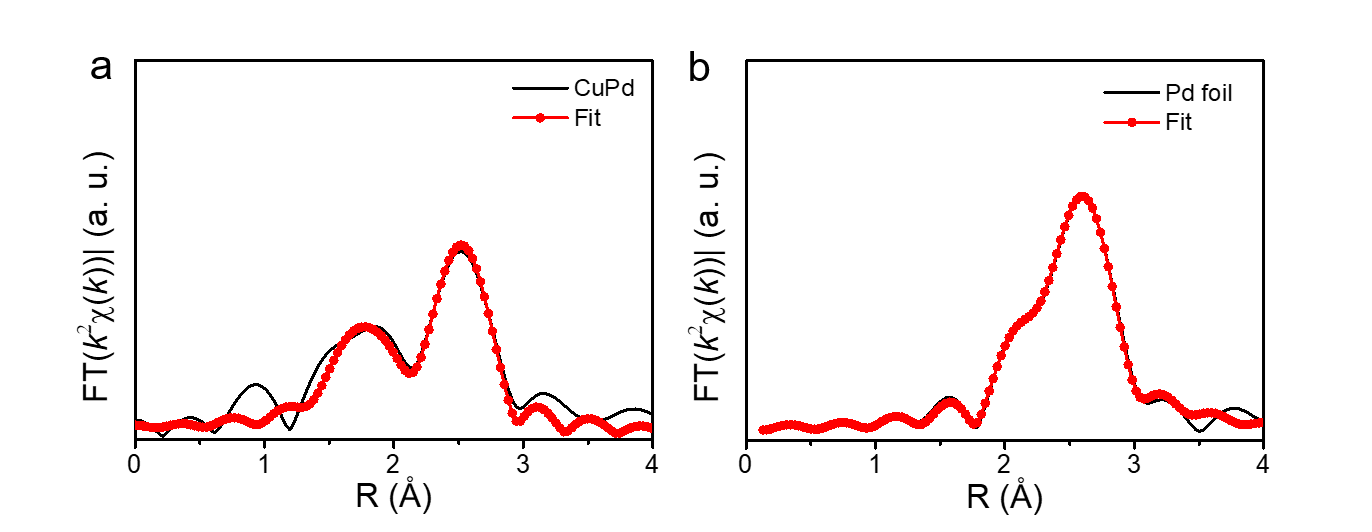


**Supplementary Figure 20 | XAFS spectra.** **a, b,** The EXAFS fitting results of the Pd K-edge for ordered CuPd nanocubes and Pd foil, respectively.

**Supplementary Table 5 |** EXAFS data fitting results of ordered CuPd nanocubes and Pd foil.

| **Sample** | **CN** | **R (Å)** | **σ^2^** | **R-factor** |
| --- | --- | --- | --- | --- |
| **CuPd nanocubes** | 7.02 ± 1.1 | 2.62 ± 0.02 | 0.0063 | 0.019 |
| **Pd foil** | 12 | 2.74 ± 0.03 | 0.0049 | 0.003 |


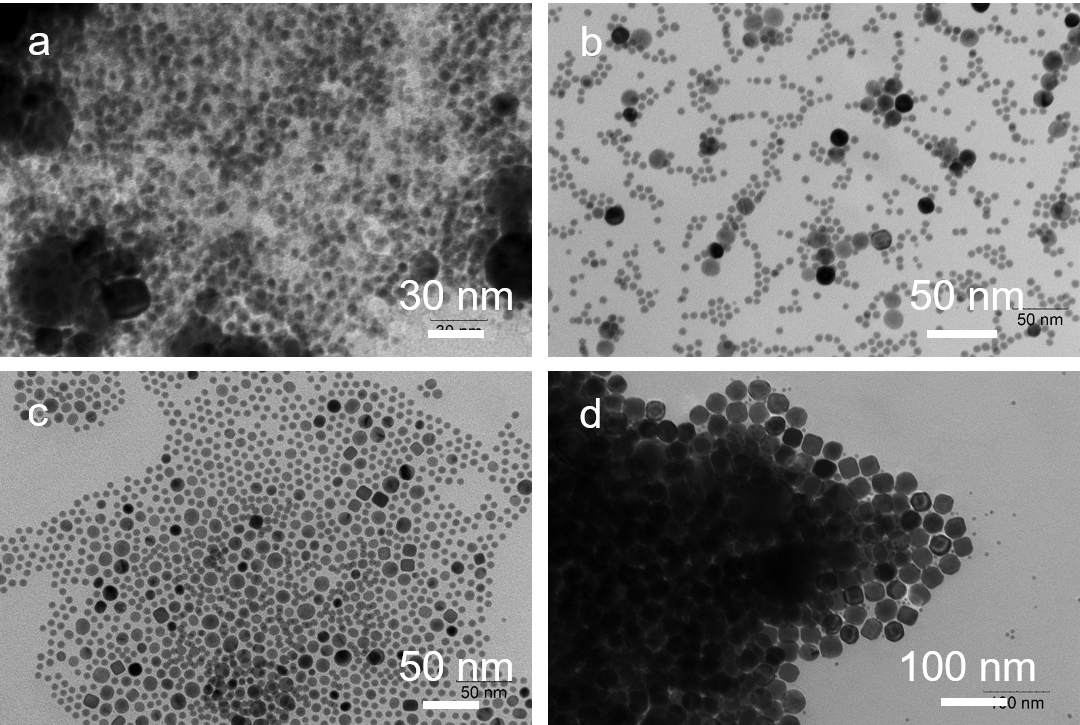


**Supplementary Figure 21 | TEM images.** TEM images of the products synthesized at 250 ^o^C for different reaction times: **a**, 1 min; **b**, 5 min; **c**, 10 min and **d**, 20 min.

**Supplementary Table 6 |** ICP results of the samples obtained at different reaction times.

| **Reaction time** | **Pd (%)** | **Cu (%)** |
| --- | --- | --- |
| **1 min** | 17.07 | 82.93 |
| **5 min** | 25.95 | 74.05 |
| **10 min** | 40.45 | 59.55 |
| **20 min** | 49.54 | 50.46 |
| **30 min** | 50.35 | 49.65 |


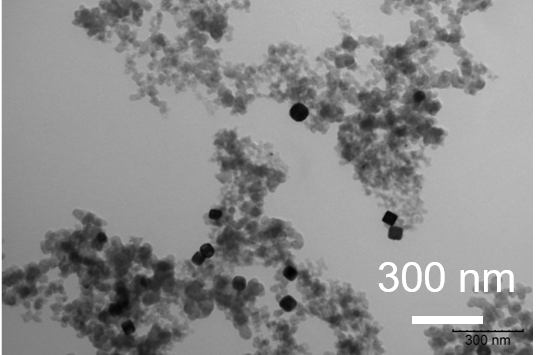


**Supplementary Figure 22 | TEM image.** **a**, A TEM image of carbon-supported ordered CuPd nanocubes.


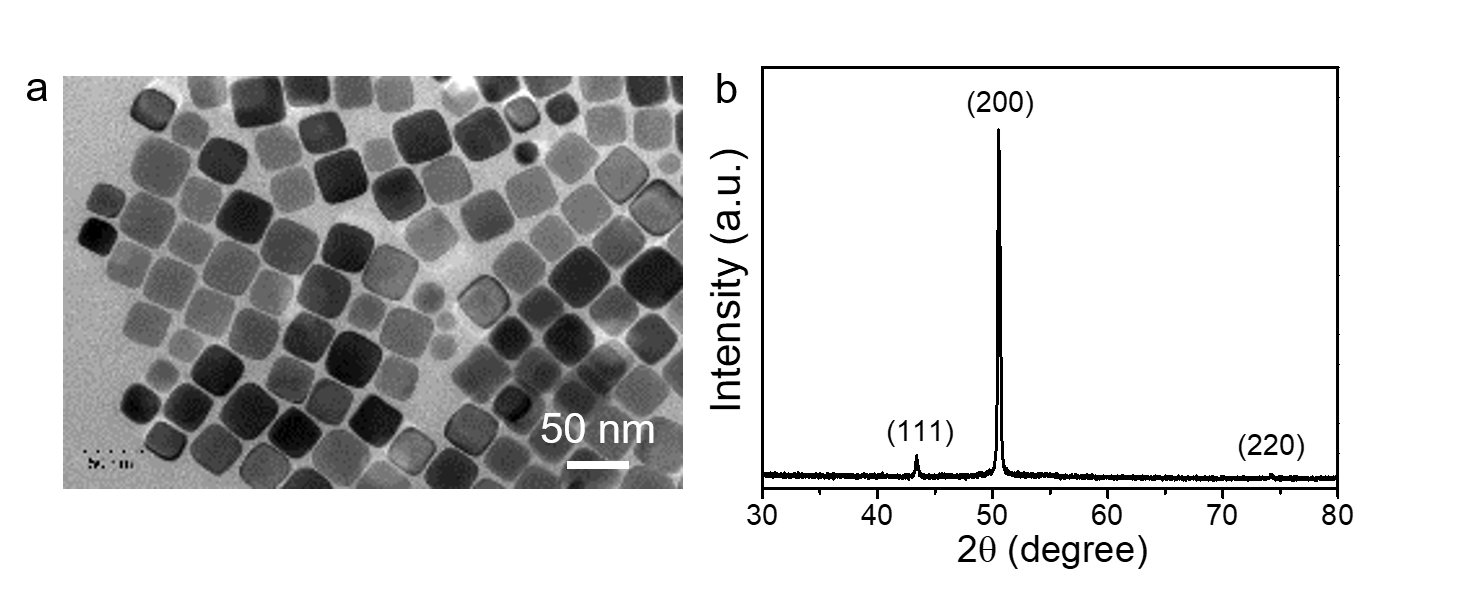


**Supplementary Figure 23 | Characterization of Cu nanocubes.** **a**, A TEM image of Cu nanocubes. **b**, The XRD pattern of Cu nanocubes.


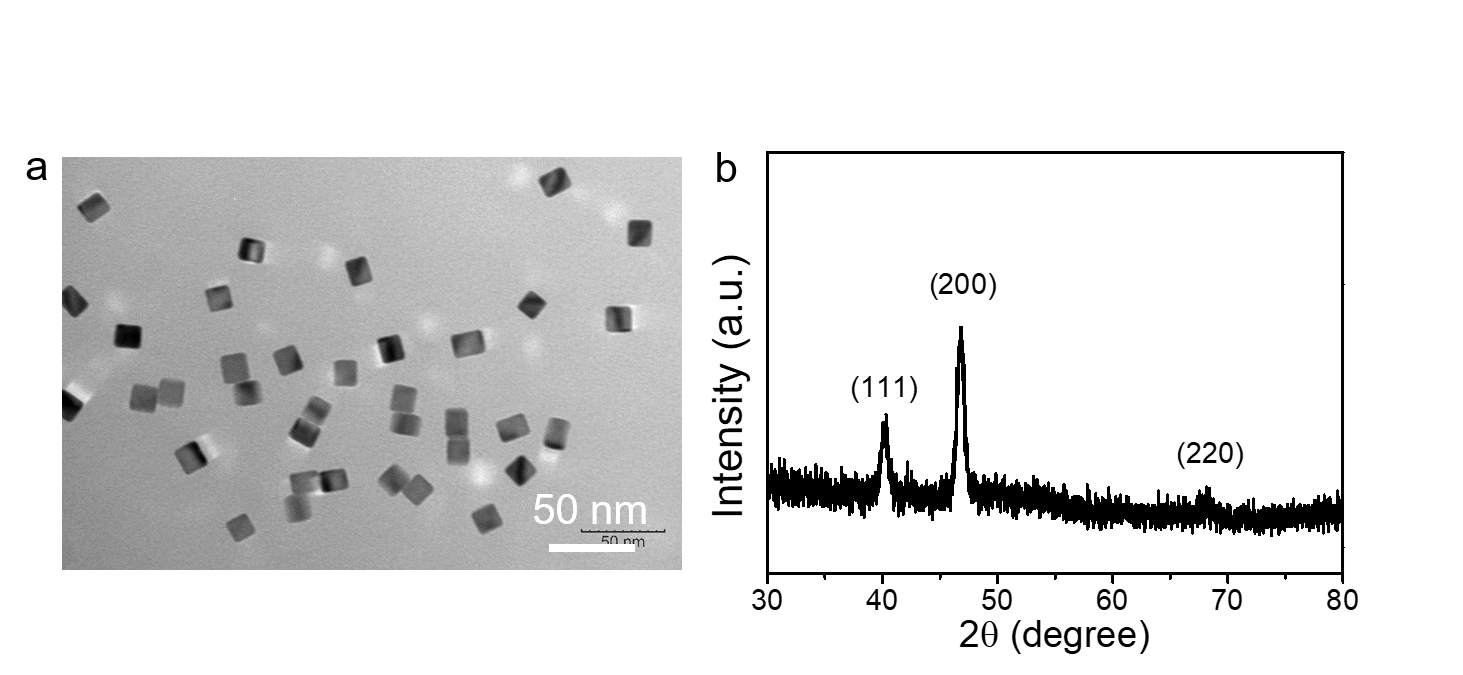


**Supplementary Figure 24 | Characterization of Pd nanocubes.** **a**, A TEM image of pure Cu nanocubes. **b**, The XRD pattern of Pd nanocubes.

**Supplementary Figure 25 | Electrocatalytic HER performance.** LSV curves of ordered CuPd nanocubes, Cu nanocubes, and Pd nanocubes normalized to the geometric area. The polarization curves were obtained by sweeping the potential from -0.5 to -1.7 V vs. Ag/AgCl with a sweep rate of 20 mV s^-1^ in 1 M KOH.


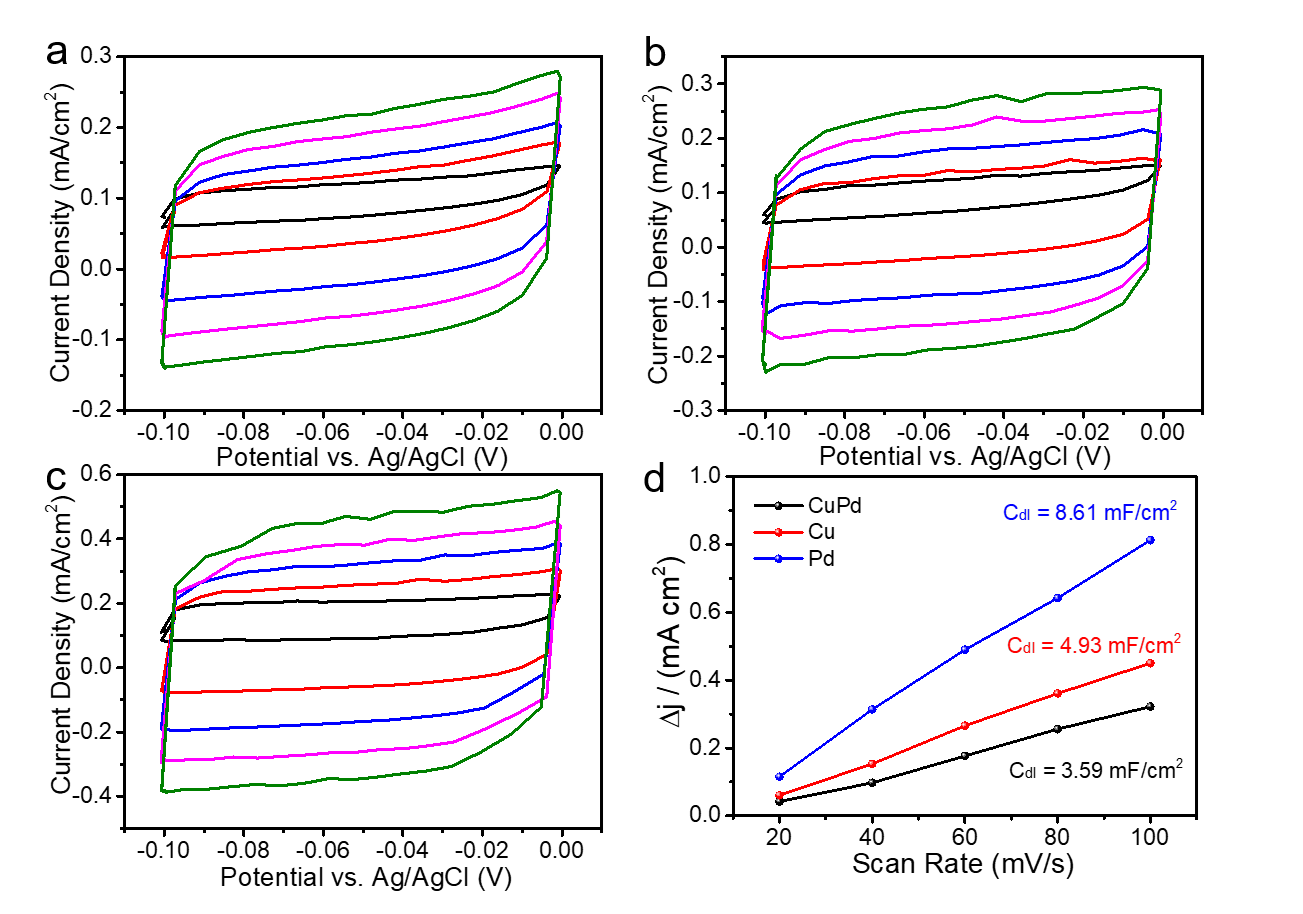


**Supplementary Figure 26 |** **Capacitance measurement**. Cyclic voltammograms in the region of -0.1-0 V vs. Ag/AgCl for different studied samples: **a**, ordered CuPd nanocubes; **b**, Cu nanocubes; and **c**, Pd nanocubes. **d**, Plots showing the extraction of the *C*_dl_ for different studied samples.


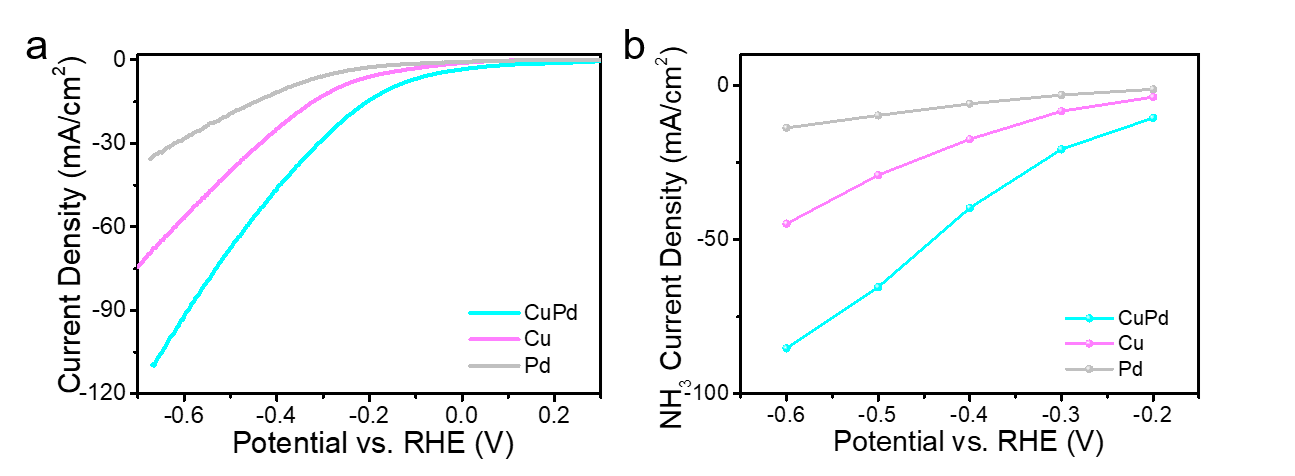


**Supplementary Figure 27 |** **Electrocatalytic NO_3_RR performance**. **a**, LSV curves of ordered CuPd nanocubes, Cu nanocubes, and Pd nanocubes normalized to ECSA. **b**, Partial NH_3_ current densities normalized to ECSA.


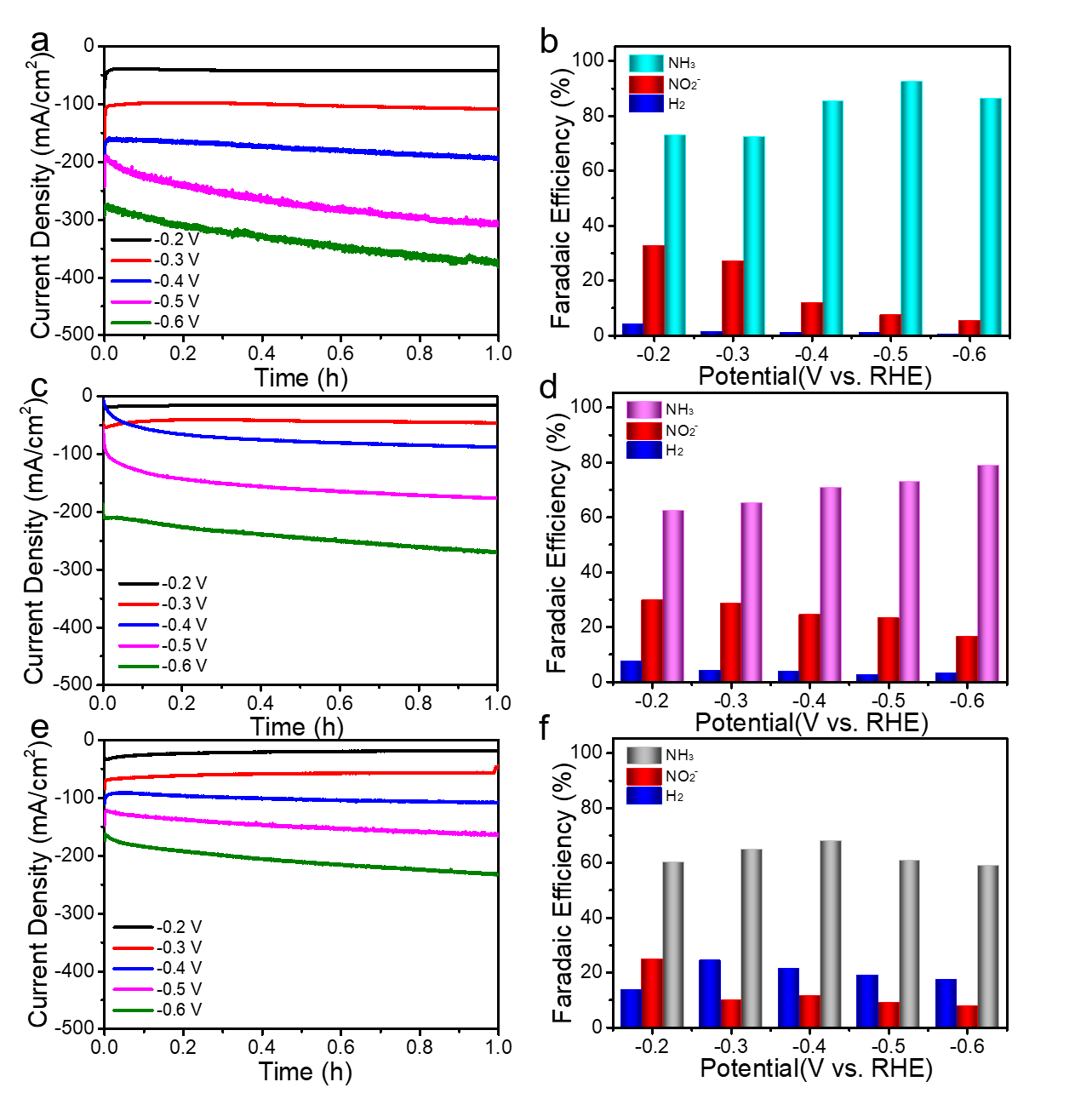


**Supplementary Figure 28 |** **Electrocatalytic NO_3_RR performance**. CA measurements of ordered CuPd nanocubes at different potentials for 1 h in 1 M KOH + 1 M KNO_3_ solution normalized to geometric area. **a,** ordered CuPd nanocubes; **c,** Cu nanocubes; **e,** Pd nanocubes. Faradaic efficiency of products at different potentials. **b**, ordered CuPd nanocubes; **d,** Cu nanocubes; **f,** Pd nanocubes.


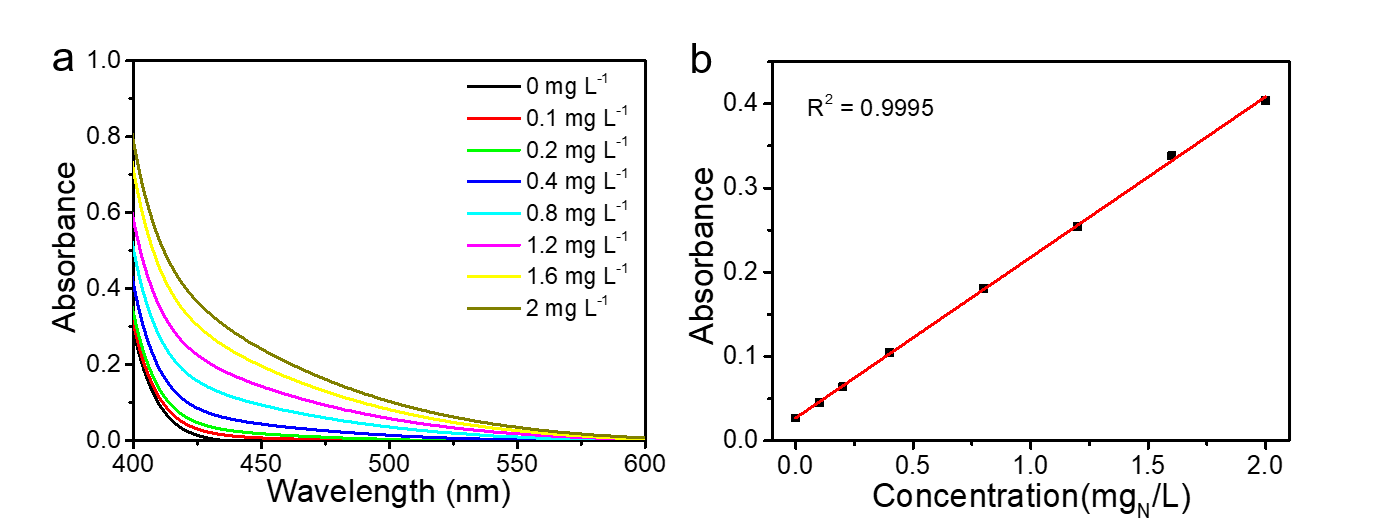


**Supplementary Figure 29 |** **UV-vis measurements of the concentrations of ammonium with Nessler’s reagents.** **a,** The ultraviolet-visible adsorption spectra of different solutions with different ammonium concentrations. **b,** Calibration curve colorimetric NH_3_ assay using Nessler reagent. Electrolytes after catalysis were diluted to ensure the ammonia concentrations in the test solutions were in the linear range of the calibration curve.

**Supplementary Figure 30 |** **Ion chromatography measurements of the concentrations of NO_2_^-^.** Calibration curve of NO_2_^-^ with different concentrations. Electrolytes after catalysis were diluted to ensure the NO_2_^-^ concentrations in the test solutions were in the linear range of the calibration curve.

**Supplementary Table 7 |** Comparison of catalytic parameters of different NO_3_RR electrocatalysts.

| **Catalyst** | **Electrolyte** | **NH_3_ FE(%)** | **NH_3_ yield rate (mol/g_cat_*h)** | **Ref.** |
| --- | --- | --- | --- | --- |
| **CuPd nanocubes** | 1 M KOH + 1 M KNO_3_ | 92.5 | 6.25 | This work |
| **Cu nanocubes** | 1 M KOH + 1 M KNO_3_ | 78.9 | 4.89 | This work |
| **Pd nanocubes** | 1 M KOH + 1 M KNO_3_ | 58.2 | 3.95 | This work |
| **Fe single atom/C** | 0.5 M KNO_3_ + 0.1 M K_2_SO_4_ | 75 | 1.18 | Ref. ^10^ |
| **Oxygen-doped-Ru** | 1 M KOH + 1 M KNO_3_ | 96 | 5.56 | Ref. ^11^ |
| **Cu_50_Ni_50_ alloys** | 1 M KOH + 0.1 M KNO_3_ | 99 | N/A | Ref. ^12^ |
| **Pd_4_Cu_4_@N-pC** | N/A | <20 | N/A | Ref. ^13^ |
| **Cu/Cu_2_O** | 200 ppm NO_3_^-^-N + 0.5 M Na_2_SO_4_ | 95.8 | 0.2449 mmol ^h-1^  cm^-2^ | Ref. ^14^ |
| **TiO_2-x_ nanotubes** | 50 ppm NO_3_^-^-N + 0.5 M Na_2_SO_4_ | 85 | N/A | Ref. ^15^ |
| **Co_3_O_4-_TiO_2_/Ti** | 50 ppm NO_3_^-^-N + 0.1 M Na_2_SO_4_ | 89 | N/A | Ref. ^16^ |


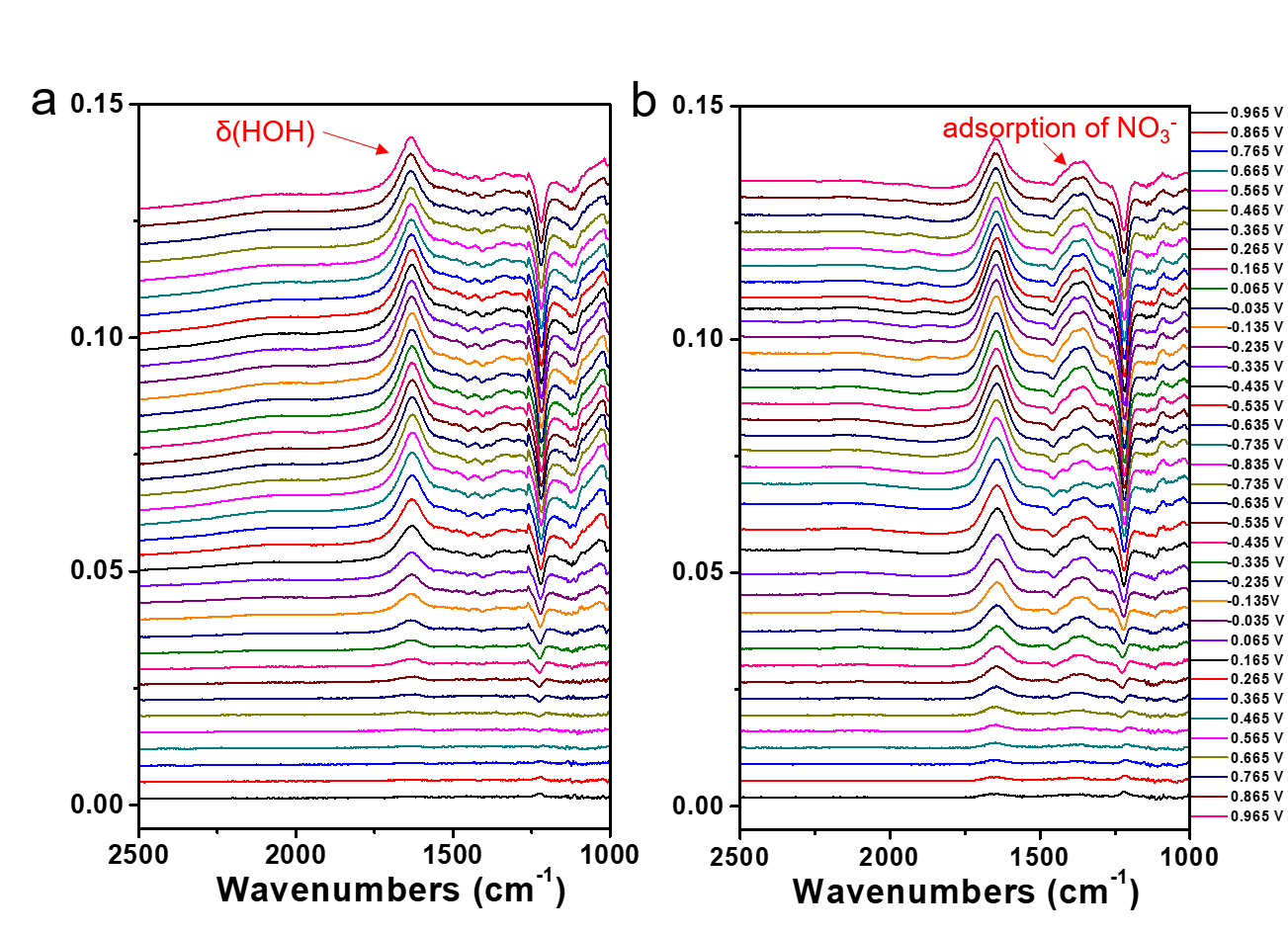


**Supplementary Figure 31 |** **ATR-SEIRAS test**. ATR-SEIRAS spectra of ordered CuPd nanocubes during CV cycling between -0.835 V and 0.965 V vs. RHE at 5 mV/s in **a**, 0.1 M KOH and **b**, 0.1 M KOH + 1 M KNO_3_.

ATR-SEIRAS experiments were conducted in a one compartment PEEK spectroelectrochemical cell with the VeeMAXIII ATR accessory. A graphite rod counter electrode and a saturated Ag/AgCl reference electrode controlled by a Metrohm Autolab potentiostat were used. IR measurements were performed in a Thermo Nicolet iS50 FTIR equipped with a liquid nitrogen cooled MCT detector. A modified chemical deposition method was used to generate Au film electrodes on the reflecting plane of Si ATR crystal prisms cut to 60° incidence. The experiments were performed in 0.1 M KOH and 0.1 M KOH + 1 M KNO_3_. 32 scans are averaged at each potential with a sampling frequency of 20 s in a CV at 5 mV/s.


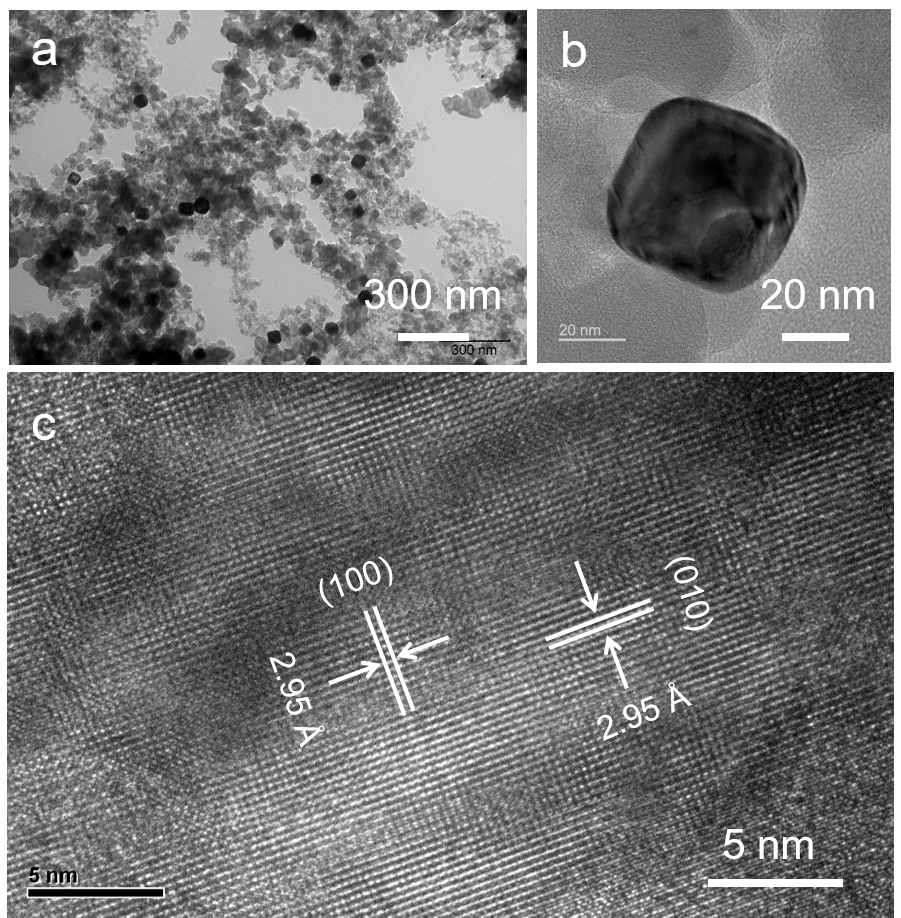


**Supplementary Figure 32 | Stability test**. **a, b,** TEM images and **c,** an HRTEM image of carbon-supported ordered CuPd nanocubes after stability test by running the CA measurement at -0.5 V vs. RHE for 12 h.

**Supplementary Figure 33 | Stability test**. XRD pattern of carbon-supported ordered CuPd nanocubes after the stability test.


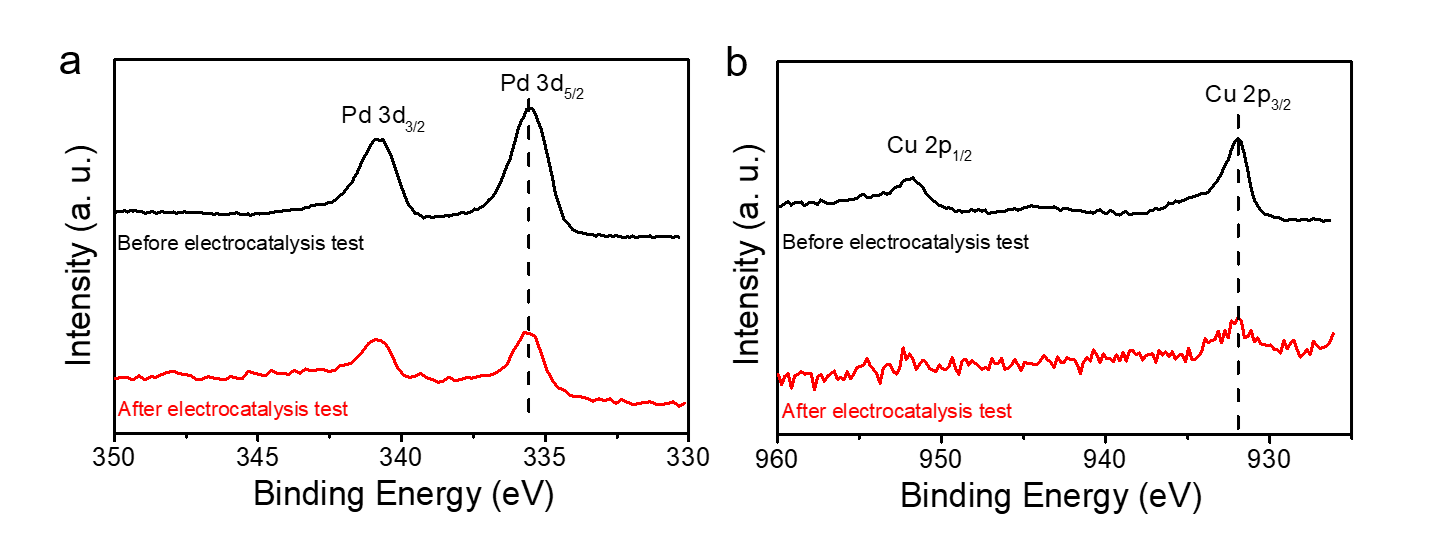


**Supplementary Figure 34 | XPS characterizations of the ordered CuPd nanocubes after stability test.** **a**, Pd 3d and **b**, Cu 2p XPS spectra for ordered CuPd nanocubes before and after the stability test.

**Supplementary Figure 35 |** **EIS Nyquist measurement**. EIS Nyquist plots of ordered CuPd nanocubes, Cu nanocubes, and Pd nanocubes. Z' is the real impedance and -Z'' is the imaginary impedance.


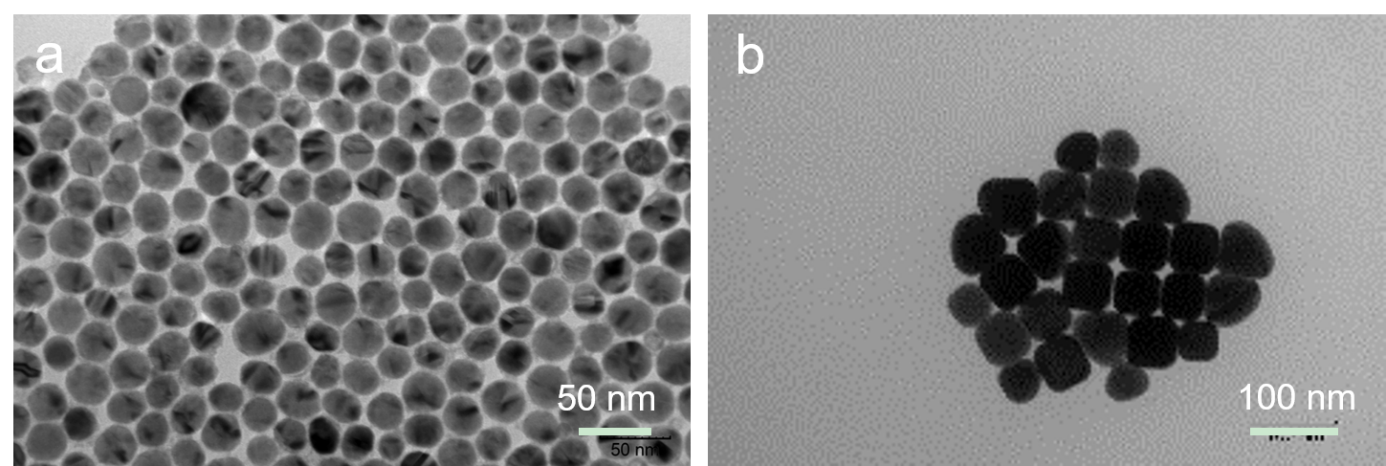


**Supplementary Figure 36 | TEM images.** **a,** TEM image of Cu nanoparticles. **b**, TEM image of Au particles (primary nanocubes). The Cu nanoparticles were synthesized by similar procedures to the synthesis of Cu nanocubes except that the precursor was changed from CuBr to Cu(acac)_2_. The Au nanocubes were synthesized according to a procedure reported previously (*Small* 2008, 4, 2059-2066).


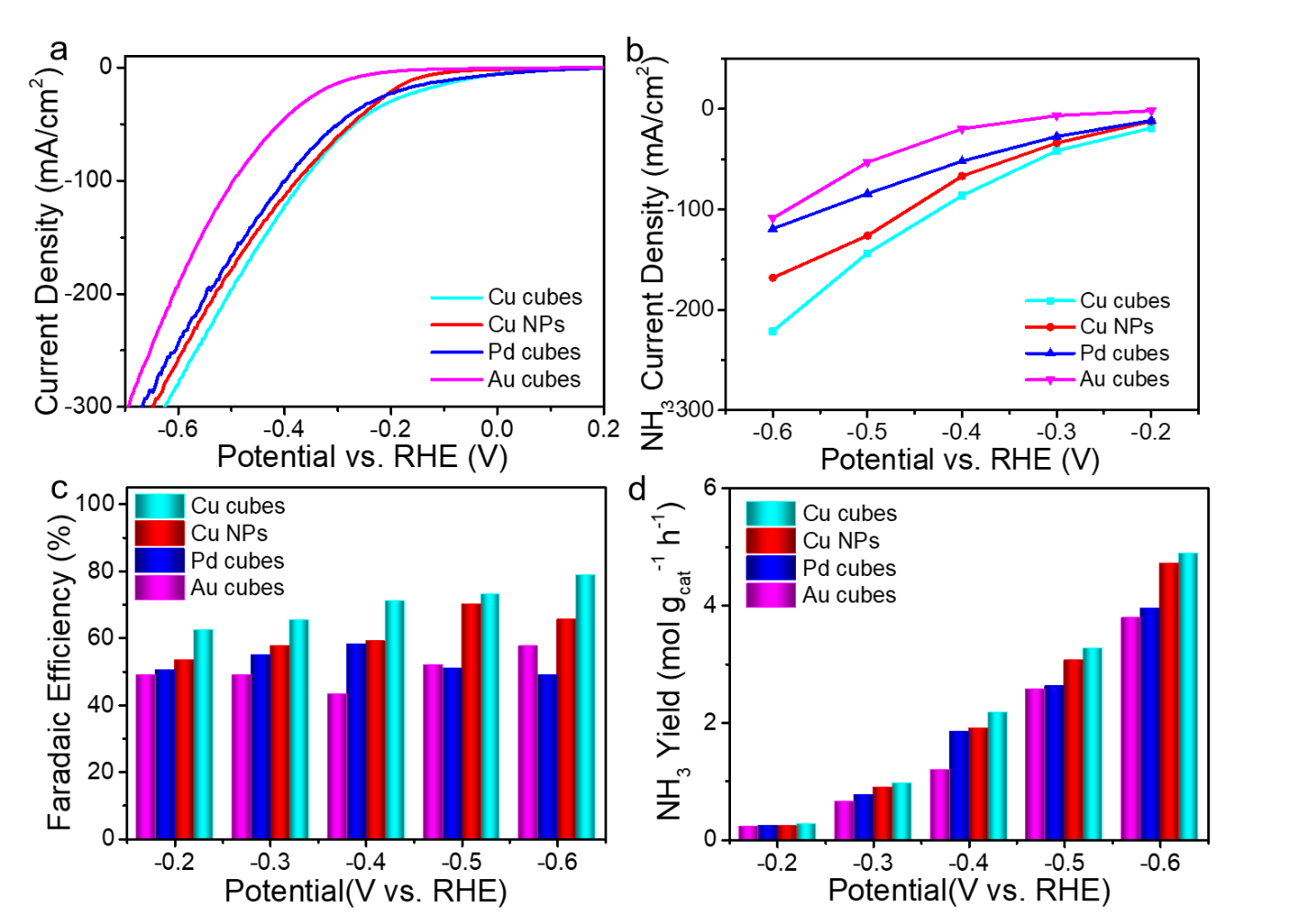


**Supplementary Figure 37 | Electrocatalytic NO_3_RR performance.** **a**, LSV curves of Cu nanocubes, Cu nanoparticles, Pd nanocubes and Au nanocubes normalized to the geometric area. **b**, Partial NH_3_ current densities normalized to the geometric area. **c**, FE of NH_3_ at different potentials. **d**, NH_3_ yield rates at different potentials.

**References**

1 Nørskov, J. K. *et al.* Origin of the Overpotential for Oxygen Reduction at a Fuel-Cell Cathode. *J. Phys. Chem. B* **108**, 17886-17892, (2004).

2 Choueiri, R. M., Tatarchuk, S. W., Klinkova, A. & Chen, L. D. Mechanism of ammonia oxidation to dinitrogen, nitrite, and nitrate on β-Ni(OH)2 from first‐principles simulations. *Electrochem. Sci. Adv.* **n/a**, 2100142, (2021).

3 Liu, J.-X., Richards, D., Singh, N. & Goldsmith, B. R. Activity and Selectivity Trends in Electrocatalytic Nitrate Reduction on Transition Metals. *Acs Catal.* **9**, 7052-7064, (2019).

4 Calle-Vallejo, F., Huang, M., Henry, J. B., Koper, M. T. M. & Bandarenka, A. S. Theoretical design and experimental implementation of Ag/Au electrodes for the electrochemical reduction of nitrate. *Phys. Chem. Chem. Phys.* **15**, 3196-3202, (2013).

5 Ong, S. P. *et al.* The Materials Application Programming Interface (API): A simple, flexible and efficient API for materials data based on REpresentational State Transfer (REST) principles. *Comput. Mater. Sci.* **97**, 209-215, (2015).

6 Jain, A. *et al.* Commentary: The Materials Project: A materials genome approach to accelerating materials innovation. *APL Materials* **1**, 011002, (2013).

7 Hjorth Larsen, A. *et al.* The atomic simulation environment-a Python library for working with atoms. *Journal of Physics: Condensed Matter* **29**, 273002, (2017).

8 Ong, S. P. *et al.* Python Materials Genomics (pymatgen): A robust, open-source python library for materials analysis. *Comput. Mater. Sci.* **68**, 314-319, (2013).

9 Wang, S., Pillai, H. S. & Xin, H. Bayesian learning of chemisorption for bridging the complexity of electronic descriptors. *Nat. Commun.* **11**, 6132, (2020).

10 Wu, Z.-Y. *et al.* Electrochemical ammonia synthesis via nitrate reduction on Fe single atom catalyst. *Nat. Commun.* **12**, 2870, (2021).

11 Li, J. *et al.* Efficient Ammonia Electrosynthesis from Nitrate on Strained Ruthenium Nanoclusters. *J. Am. Chem. Soc.* **142**, 7036-7046, (2020).

12 Wang, Y. *et al.* Enhanced nitrate-to-ammonia activity on copper-nickel alloys via tuning of intermediate adsorption. *J. Am. Chem. Soc.* **142**, 5702-5708, (2020).

13 Chen, M. *et al.* Achieving high-performance nitrate electrocatalysis with PdCu nanoparticles confined in nitrogen-doped carbon coralline. *Nanoscale* **10**, 19023-19030, (2018).

14 Wang, Y., Zhou, W., Jia, R., Yu, Y. & Zhang, B. Unveiling the Activity Origin of a Copper-based Electrocatalyst for Selective Nitrate Reduction to Ammonia. *Angew. Chem., Int. Ed.* **59**, 5350-5354, (2020).

15 Jia, R. *et al.* Boosting Selective Nitrate Electroreduction to Ammonium by Constructing Oxygen Vacancies in TiO_2_. *Acs Catal.* **10**, 3533-3540, (2020).

16 Gao, J. *et al.* Non-precious Co_3_O_4_-TiO_2_/Ti cathode based electrocatalytic nitrate reduction: Preparation, performance and mechanism. *Appl. Catal. B: Environ.* **254**, 391-402, (2019).
